# Supplementary material for: Young SINEs in pig genomes impact gene regulation, genetic diversity, and complex traits
Source: Commun Biol. 2023 Aug 31;6:894. doi: 10.1038/s42003-023-05234-x (PMC10471783; doi:10.1038/s42003-023-05234-x)
Supplement: Supplementary file 2 — Supplementary Information [file 42003_2023_5234_MOESM2_ESM.pdf]

Supplementary information

Supplementary Figure 1. Sequence composition of the consensus sequence for young SINE/PRE elements.
Supplementary Figure 2. Distribution of the bases with the highest frequencies of the consensus sequence for young SINE/PRE elements.
Supplementary Figure 3. A minimum spanning tree for 90 young SINE/PRE subfamilies.
Supplementary Figure 4. Sequence divergence distribution and total sizes of the 17 new large SINE/PRE subfamilies.
Supplementary Figure 5. Sequence divergence distribution of four categories of SINE families.
Supplementary Figure 6. The enrichment levels of four SINE groups in the A and B compartments.
Supplementary Figure 7. The enrichment levels of four SINE groups and CTCF in the central and boundary regions of TADs.
Supplementary Figure 8. The density of small non-coding RNAs on SINE families.
Supplementary Figure 9. Four categories of young SINE-associated transcripts.
Supplementary Figure 10. Scale independence and mean connectivity for WGCNA.
Supplementary Figure 11. 40 co-expressed modules were derived from 13,872 PCGs.
Supplementary Figure 12. The enrichment levels of gene modules in various tissues.
Supplementary Figure 13. Network for the high correlation between gene modules and tissues.
Supplementary Figure 14. Gene-to-gene networks of topological clustering by the Markov clustering algorithm.
Supplementary Figure 15. The enrichment of SINE-associated gene modules in various tissues.
Supplementary Figure 16. The phylogenetic tree and heatmap for tissues and gene modules.
Supplementary Figure 17. The identified counts and verification rates of *Ref*- ploySINEs for different detection tools.
Supplementary Figure 18. The identified counts and verification rates of *Ref*+ ploySINEs for different detection tools.
Supplementary Figure 19. The identified counts of ploySINE for Meishan and Duroc pigs using MELT tools with different sequencing depths.
Supplementary Figure 20. Performance comparison of the detection tools for *Ref*+ ploySINEs at the sequencing depth of 5X.
Supplementary Figure 21. Performance comparison of the detection tools for *Ref*+ ploySINEs at the sequencing depth of 10X.
Supplementary Figure 22. Performance comparison of the detection tools for *Ref*+ ploySINEs at the sequencing depth of 15X.
Supplementary Figure 23. Performance comparison of the detection tools for *Ref*+ ploySINEs at the sequencing depth of 20X.
Supplementary Figure 24. Performance comparison of the detection tools for *Ref*+ ploySINEs at the sequencing depth of 30X.

Supplementary Figure 25. Performance comparison of the detection tools for *Ref*+ ploySINEs at the sequencing depth of 50X.
Supplementary Figure 26. Distribution of mapping rate and the total number of mapped bases for each individual.
Supplementary Figure 27. Density distribution of polySINEs in the whole population.
Supplementary Figure 28. Population frequency of polySINEs among different groups
Supplementary Figure 29. Population structure based on polySINEs for 381 individuals when K varied from 3 to 15.
Supplementary Figure 30. Overviews of 79 published GWAS studies involve 97 complex traits.
Supplementary Figure 31. Sankey plot represents the relationship among systems, tissues, genes, and traits.
Supplementary Figure 32. The LD block of 15kb at chr8 from 109,439,023 to 109,454,866.
Supplementary Figure 33. NCBI annotation of *VRTN* gene and the RNA-seq exon coverage on the first exon of *VRTN*.

Supplementary Figures

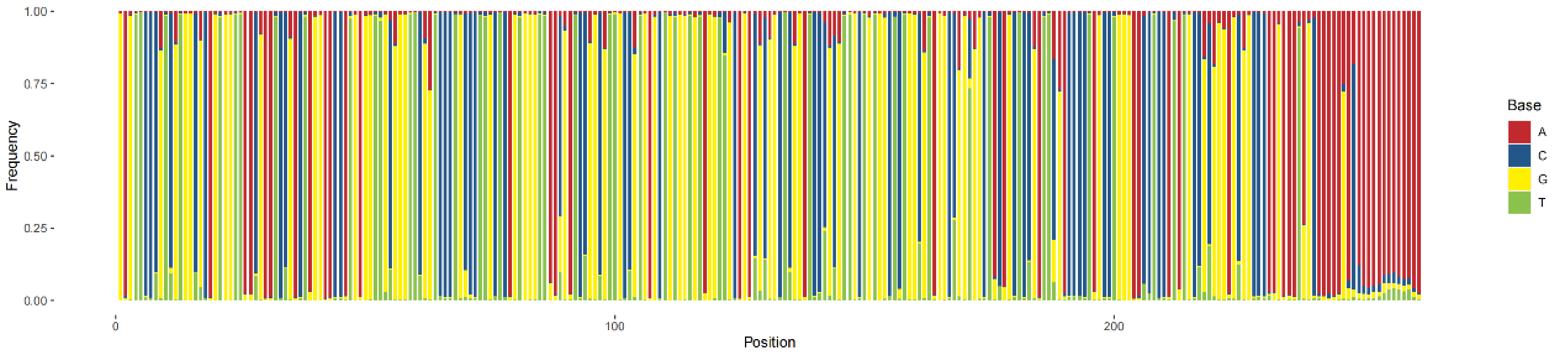

Supplementary Figure 1. Sequence composition of the consensus sequence for young SINE/PRE elements (PRE1-SS, PRE0-SS, and PRE1a). After processing all of the full-length young SINEs (PRE1-SS, PRE0-SS, and PRE1a) from 14 publicly available pig genomes, we retained 978,506 non-redundant young SINEs and created their consensus sequence by multiple sequence alignment.

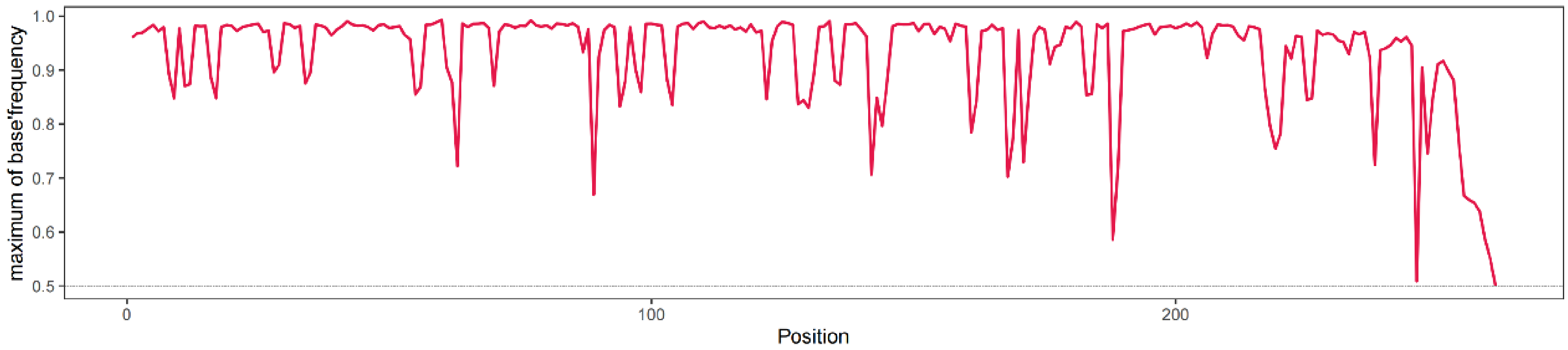

Supplementary Figure 2. Distribution of the bases with the highest frequencies of the consensus sequence for young SINE/PRE elements (PRE1-SS, PRE0-SS, and PRE1a). After processing all of the full-length young SINEs (PRE1-SS, PRE0-SS, and PRE1a) from 14 publicly available pig genomes (Supplementary Information), we retained 978,506 non-redundant young SINEs and created their consensus sequence by multiple sequence alignment.

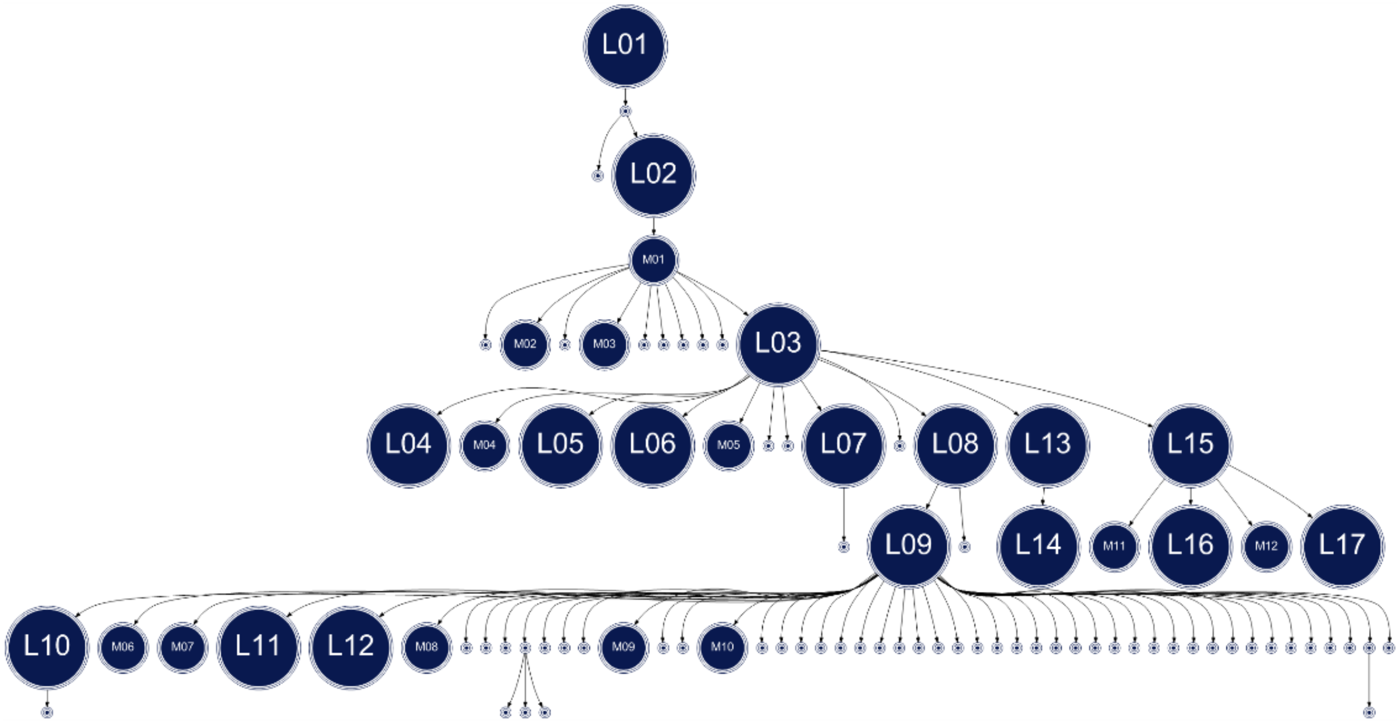

Supplementary Figure 3. A minimum spanning tree for 90 young SINE/PRE subfamilies. This minimum spanning tree analysis recategorized 978,506 non-redundant young SINE/PREs (PRE1-SS, PRE0-SS, and PRE1a) into 90 new SINE/PRE subfamilies, including 17 large (labels beginning with L, size > 10000), 12 medium (M, size ≥ 3000 and ≤ 10,000) and 61 small (S, size < 3000) SINE/PRE subfamilies.

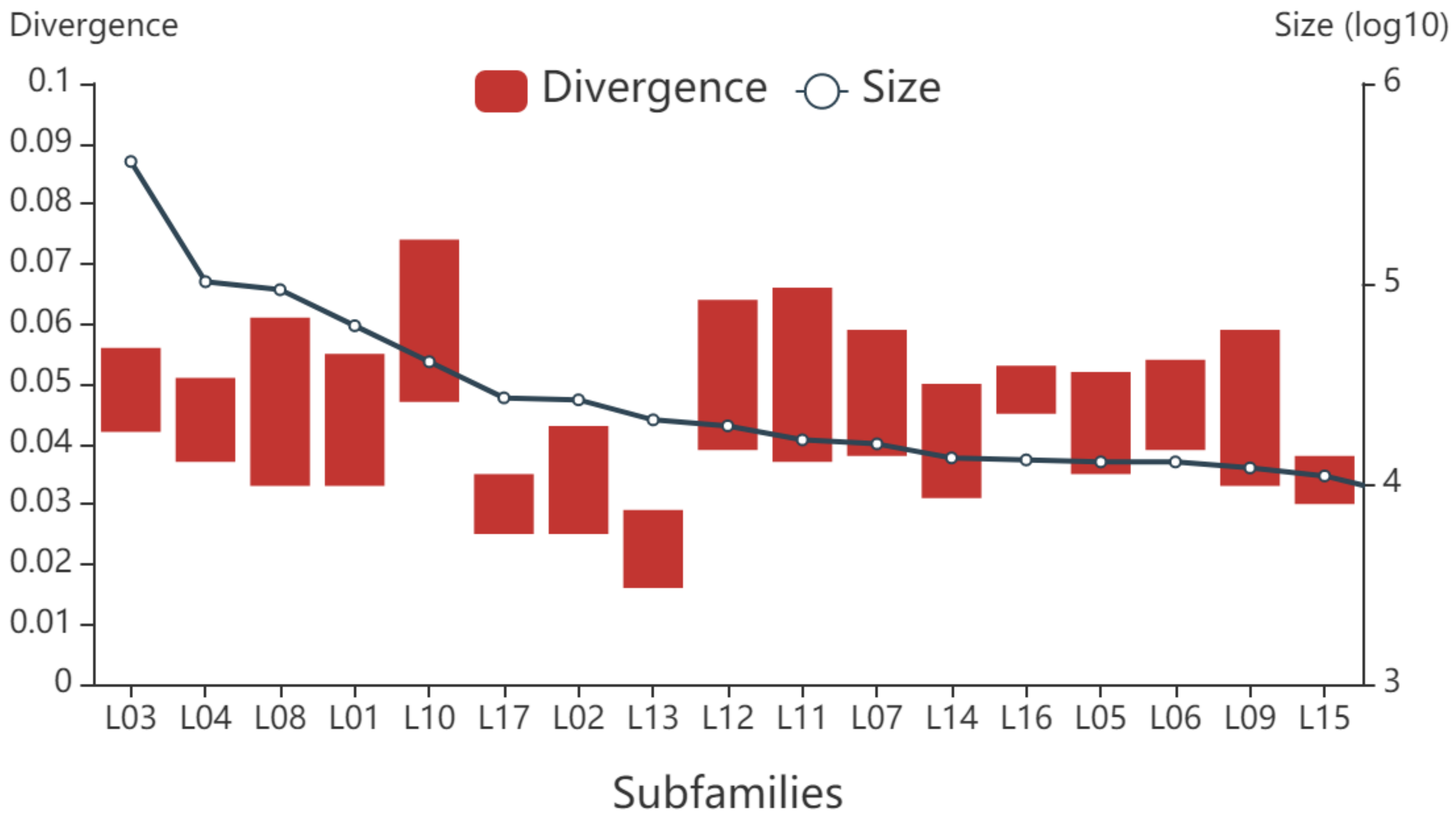

Supplementary Figure 4. Sequence divergence distribution and total sizes of the 17 new large SINE/PRE subfamilies. The left y-axis represents the divergence of large SINE/PRE subfamilies, and the right y-axis represents the counts of SINE/PRE subfamilies

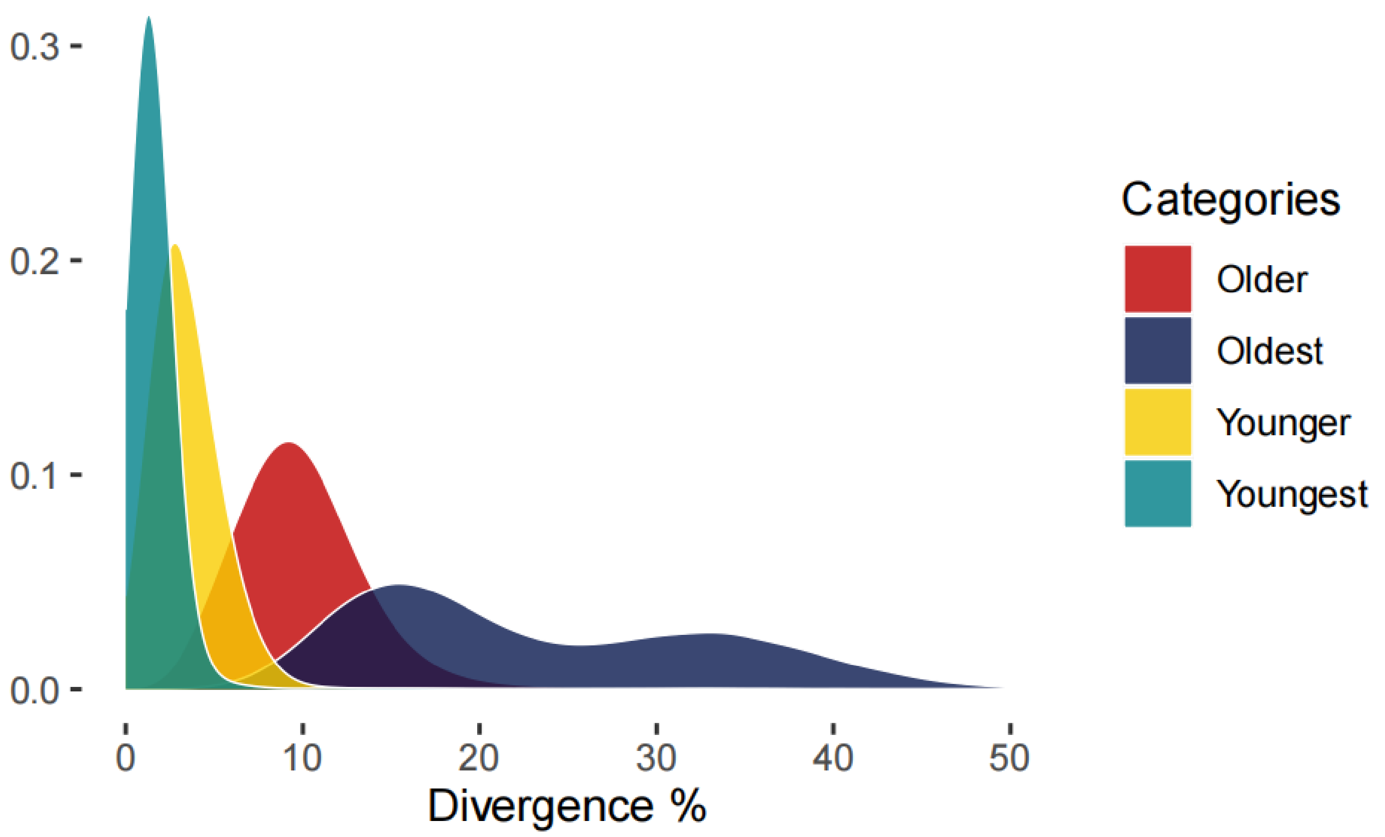

Supplementary Figure 5. Sequence divergence distribution of four categories of SINE families, including youngest (L13 subfamilies), younger (young SINE families except for L13 subfamilies, non-L13), older (non-young PRE families), and oldest (non-PRE families; ancient families) SINEs. The x-axis represents the divergence of TE and the y-axis represents the density for each TE family.

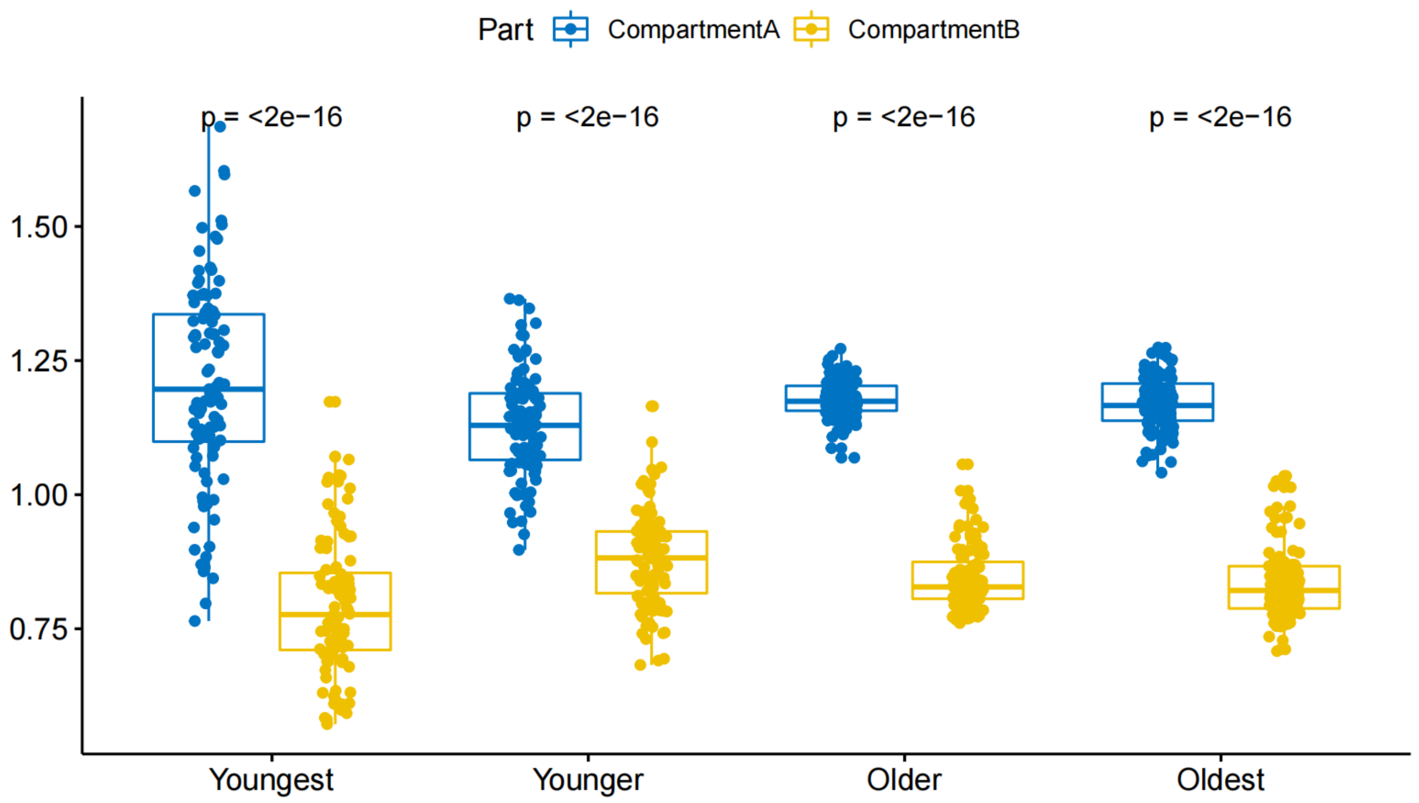

Supplementary Figure 6. The enrichment levels of four SINE groups in the A and B compartments. The x-axis represents the four SINE groups, and the y-axis represents the normalized density. The x-axis represents the TE groups, and the y-axis represents the average fold enrichment for each chromosome. The line inside the boxplots represents the median.

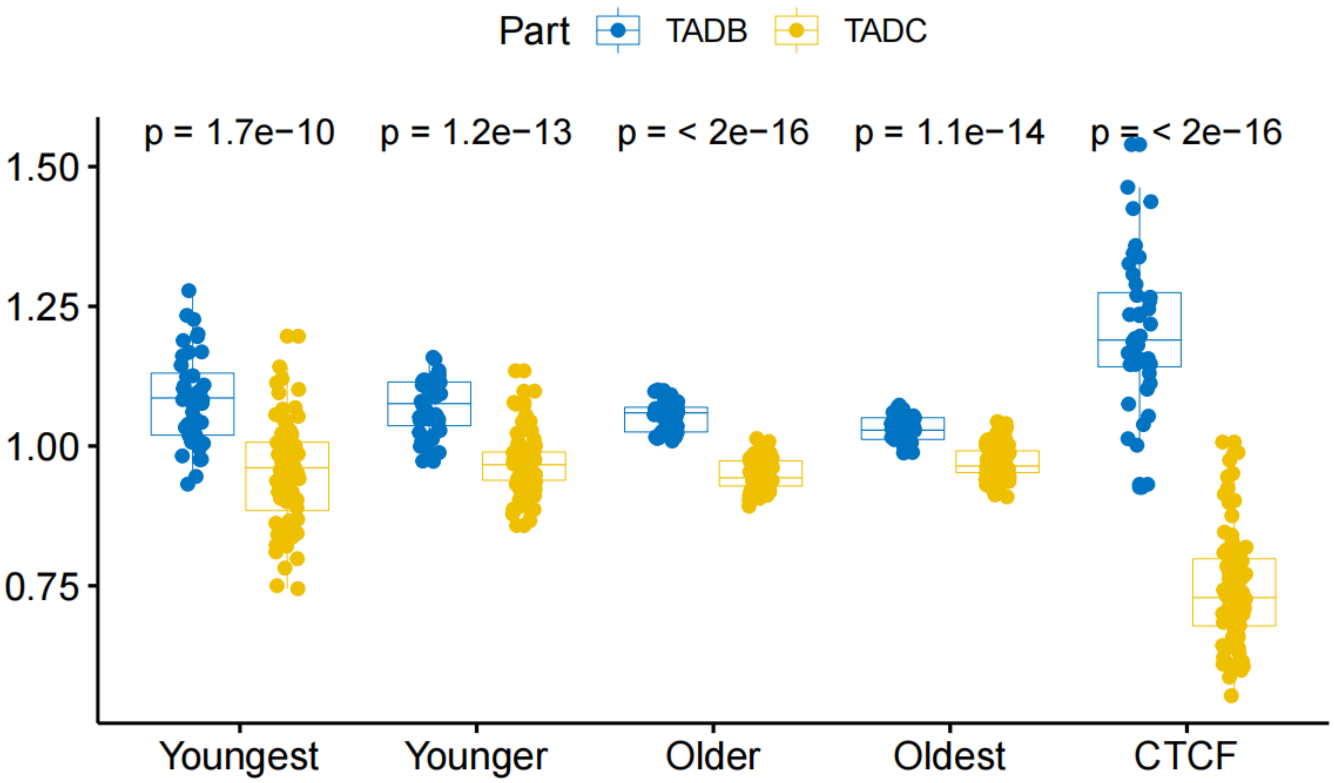

Supplementary Figure 7. The enrichment levels of four SINE groups and CTCF in the central and boundary regions of TADs. The x-axis represents the four SINE groups, and the y-axis represents the average normalized density for each chromosome. TADB and TADC represent the boundary and central regions of TADs, respectively. The line inside the boxplots represents the median.

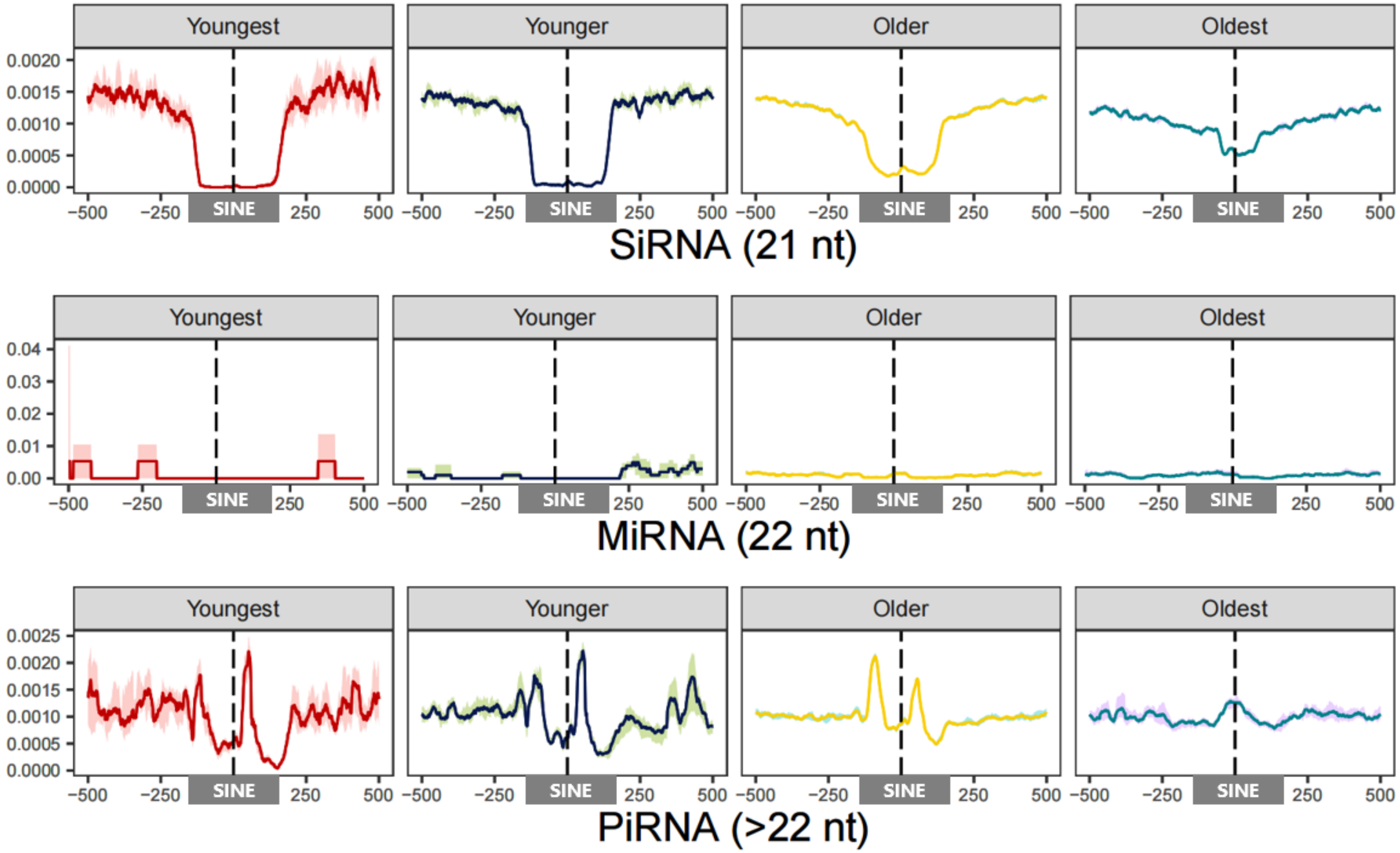

Supplementary Figure 8. The density of small non-coding RNAs on SINE families. The x-axis represents the distance to the center of TE, and the y-axis represents the enrichment signals. The shading in the figure represents the 95% confidence intervals, which are shown as error bars above and below the mean column.

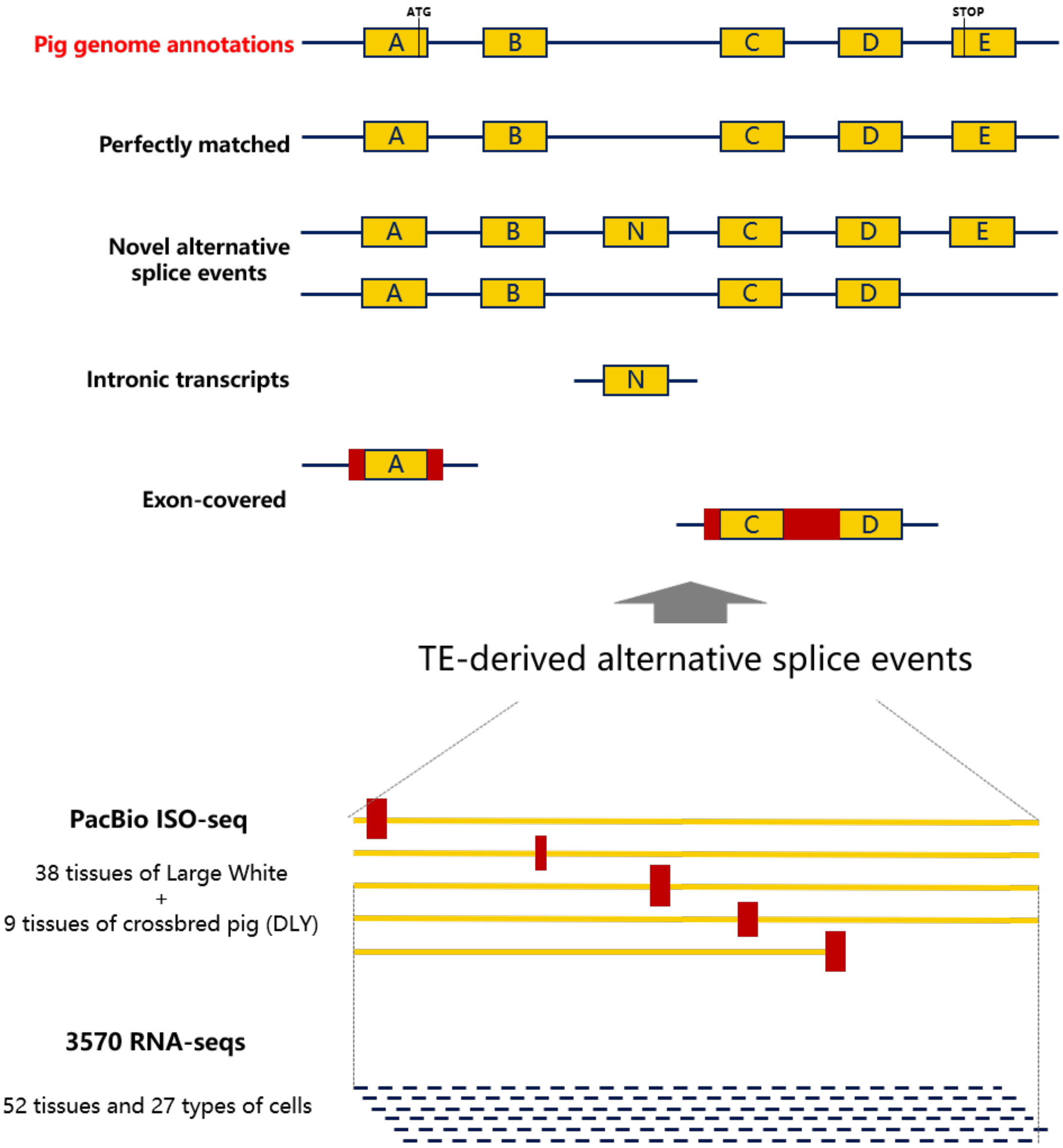

Supplementary Figure 9. Four categories of young SINE-associated transcripts. Younger and youngest SINE-associated transcripts into four categories by comparing their genomic location with known transcripts in the currently available pig genome annotations: perfectly matched, novel alternative splice events, intronic, and exon-covered transcripts.

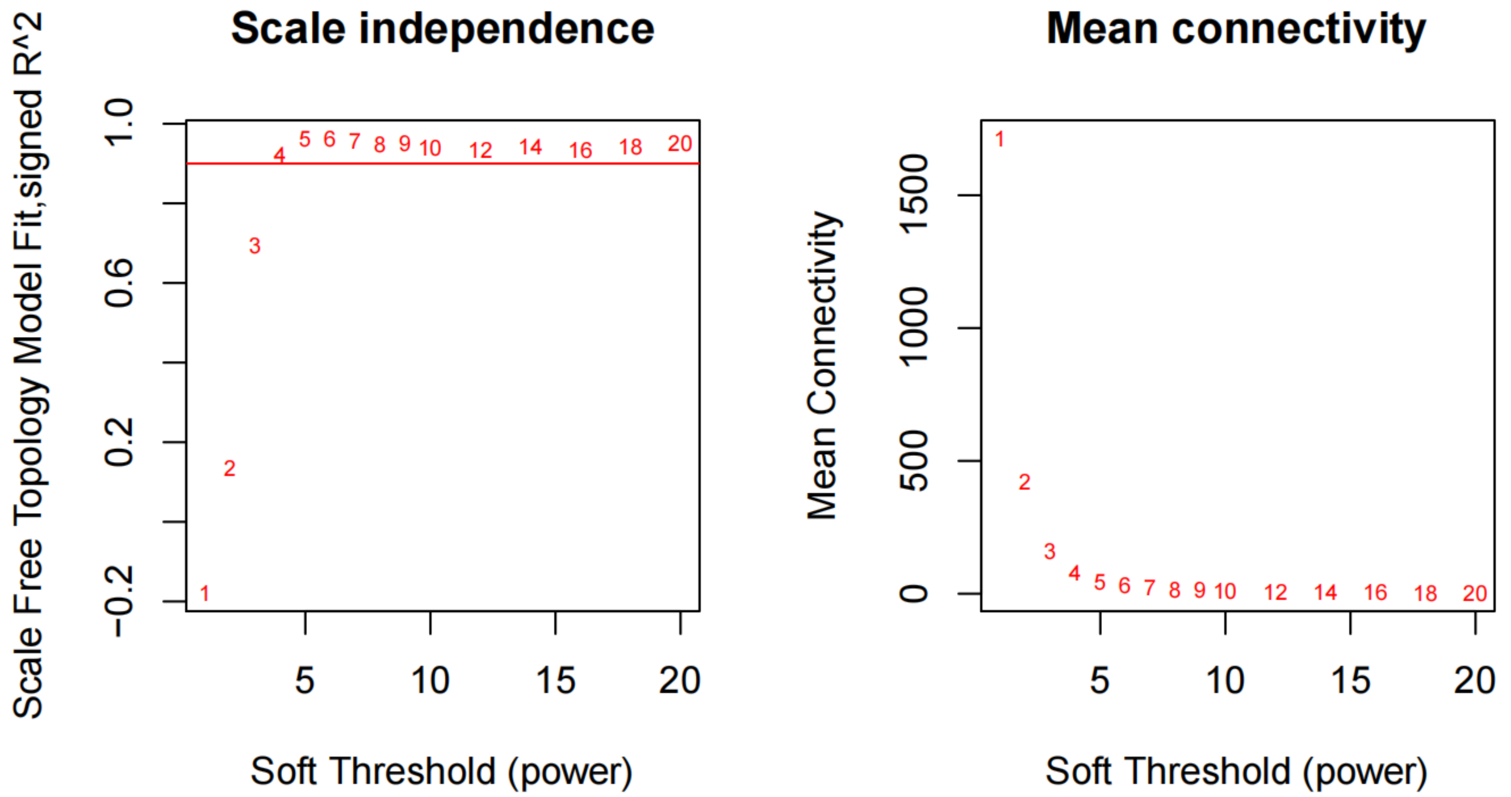

Supplementary Figure 10. Scale independence and mean network connectivity for soft thresholding powers (β). β = 4 was selected to achieve model fit maximization.

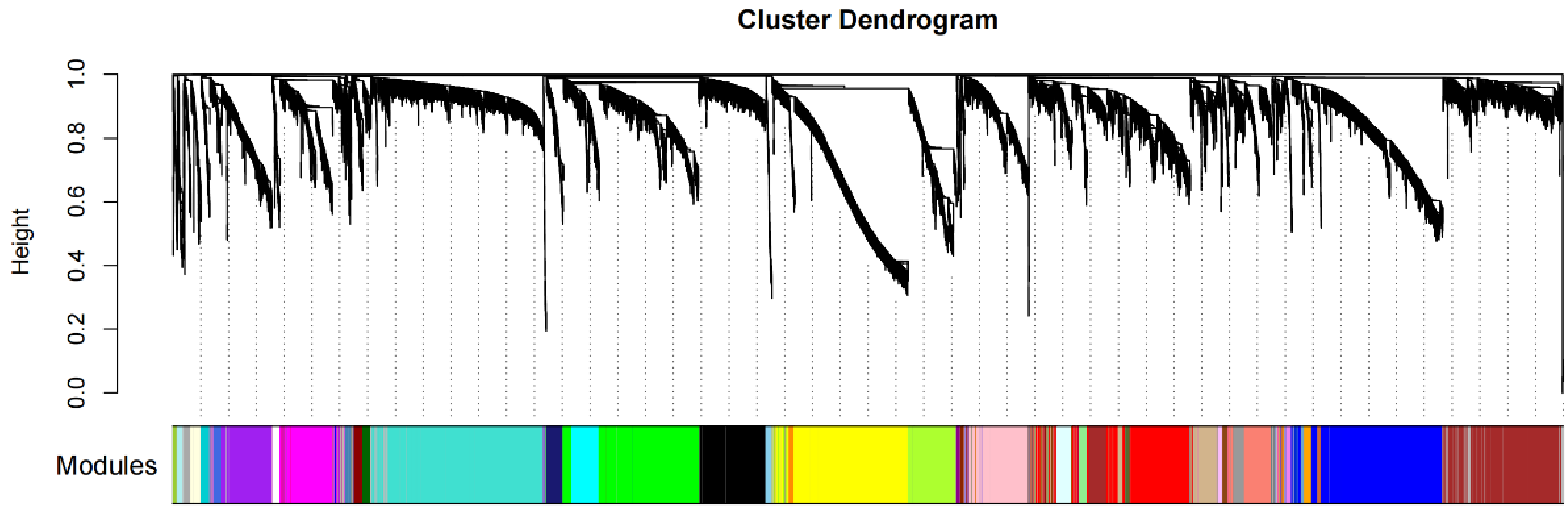

Supplementary Figure 11. 40 co-expressed modules were derived from 13,872 PCGs. The top image shows a gene dendrogram, and the bottom image shows the gene modules with different colors.

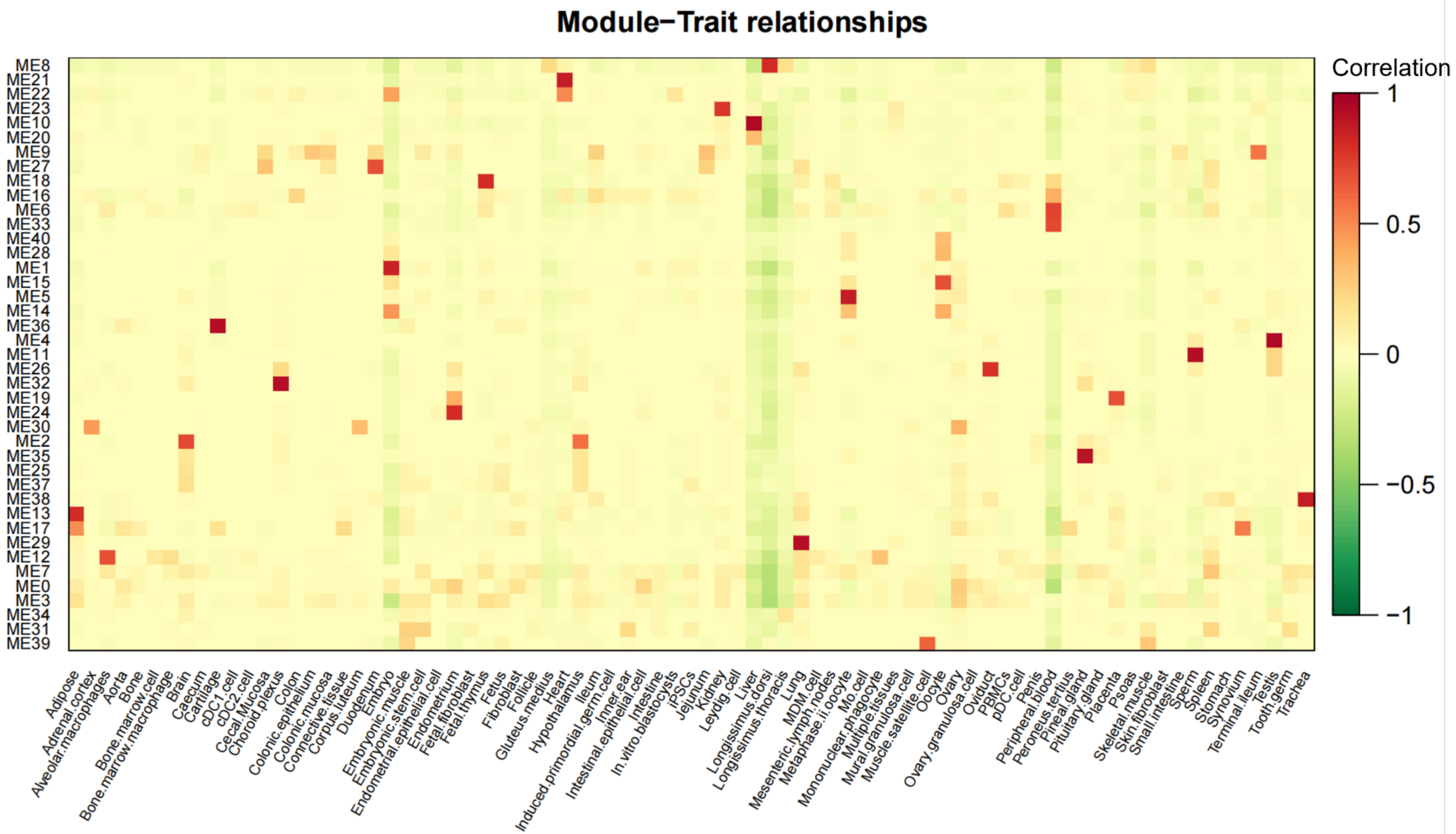

Supplementary Figure 12. The enrichment levels of gene modules in various tissues. The x-axis represents the types of tissues and cells, while the y-axis represents the gene modules. The heatmap represents the degree of enrichment between modules and tissues. Positive correlations are shown in red, while negative correlations are shown in green.

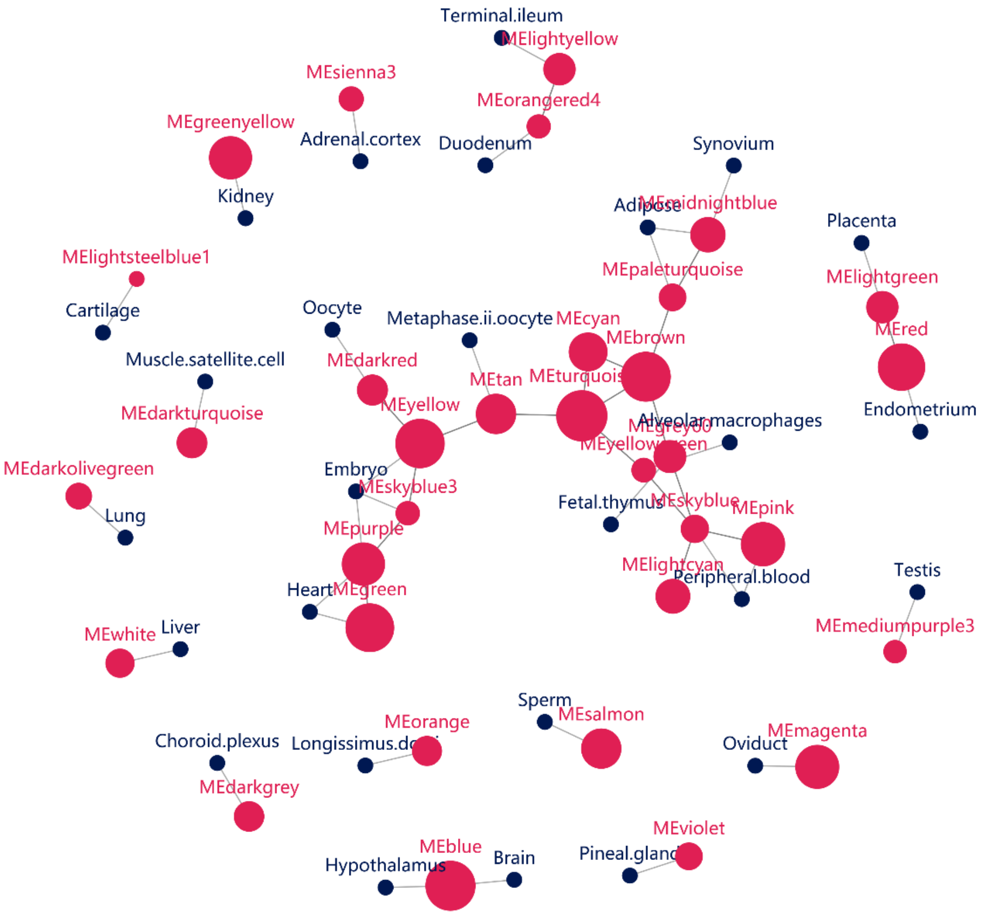

Supplementary Figure 13. Network for the high correlation between gene modules and tissues. The red spot represents the gene modules, while the blue spot represents the tissues and cells. The size of the red dot corresponds to the number of genes contained in the module.

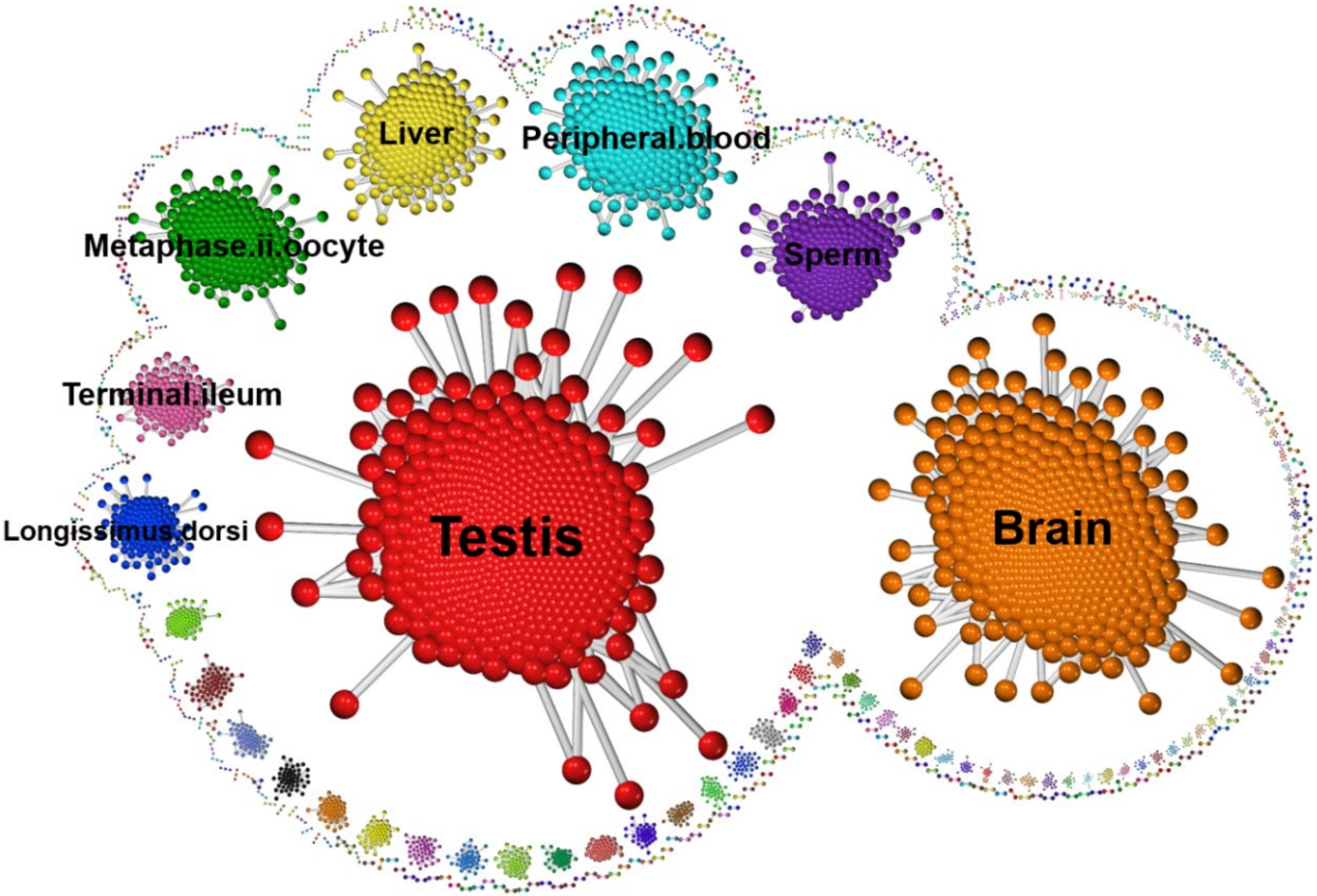

Supplementary Figure 14. Gene-to-gene networks of topological clustering by the Markov clustering algorithm. The clustering patterns can be visualized by assigning colors to groups of genes that are enriched in specific tissues.

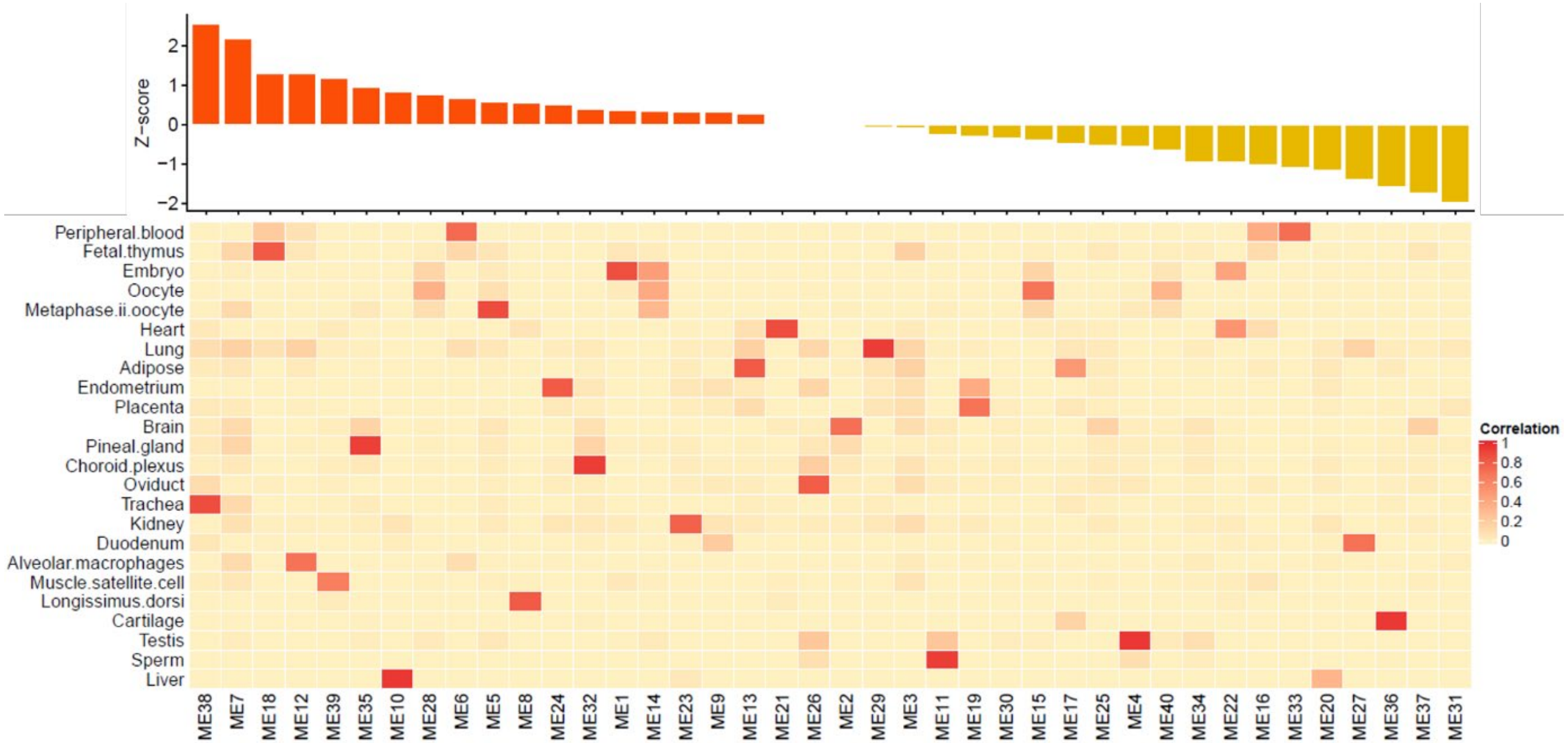

Supplementary Figure 15. The enrichment of SINE-associated gene modules in various tissues. The bars in the upper section represent the enrichment levels of SINE-associated gene modules. Below is a heat map that shows the association between modules and different types of tissues and cells.

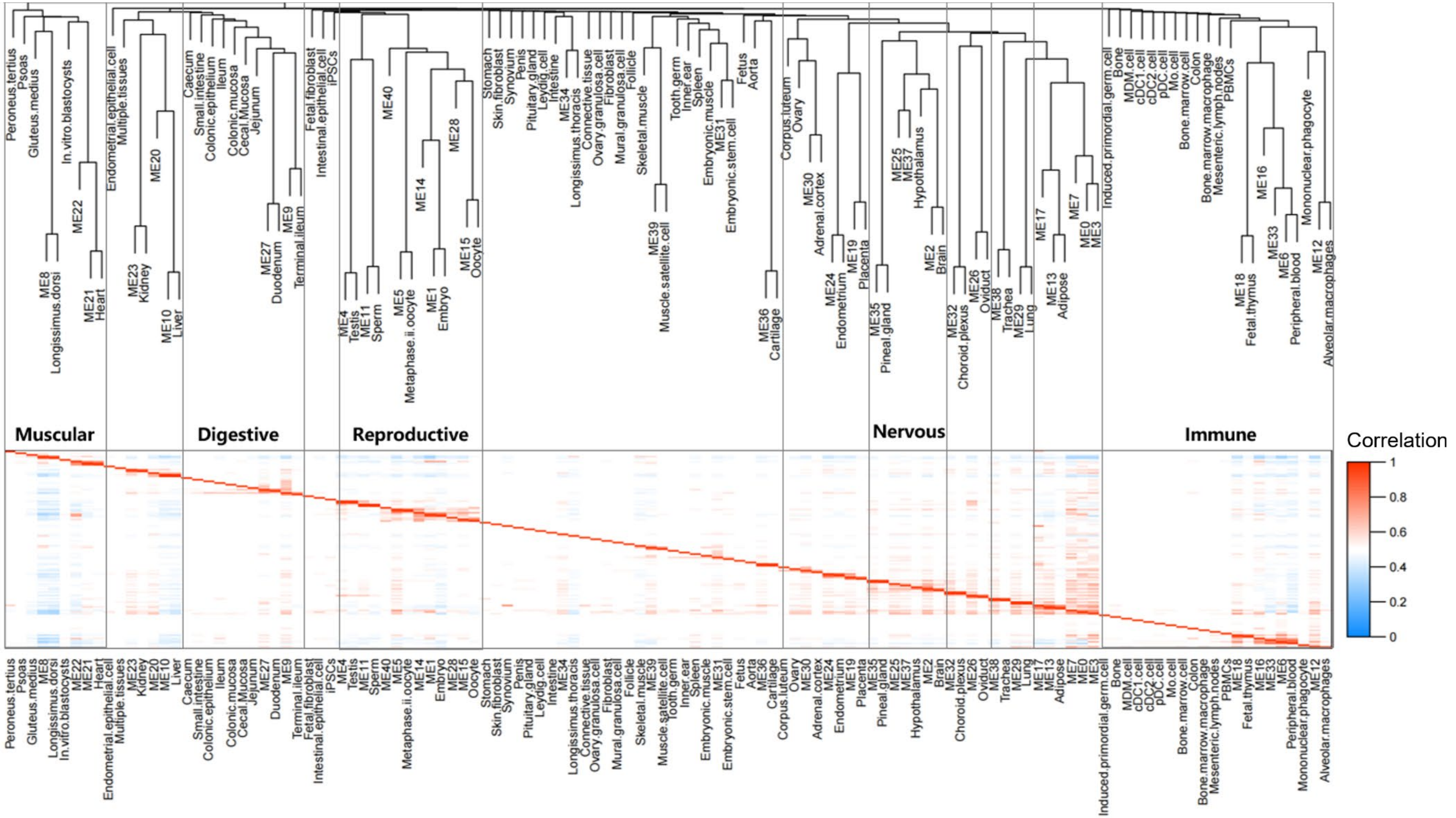

Supplementary Figure 16. The phylogenetic tree and heatmap for tissues and gene modules. The phylogenetic tree and heatmap show the clustering between gene modules and different types of tissues and cells.

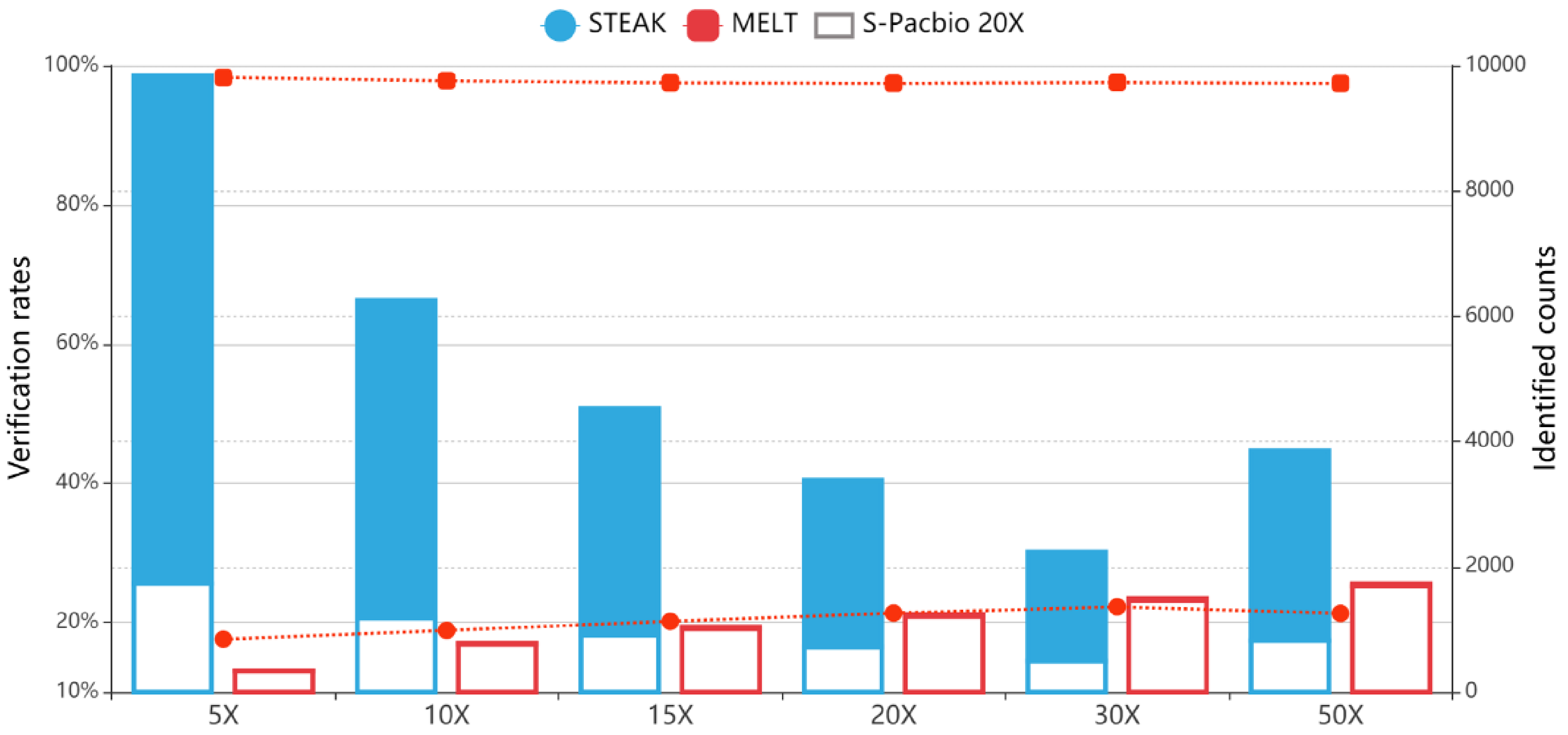

Supplementary Figure 17. The identified counts and verification rates of *Ref-* ploySINEs are shown for different detection tools at varying sequencing depths. The blue bar represents the counts identified by STEAK, while the red bar represents those identified by MELT. In both STEAK and MELT, the blank bar represents the counts that are supported by ploySINEs from Pacbio. The square and circular lines represent the validation rates of STEAK and MELT by Pacbio, respectively.

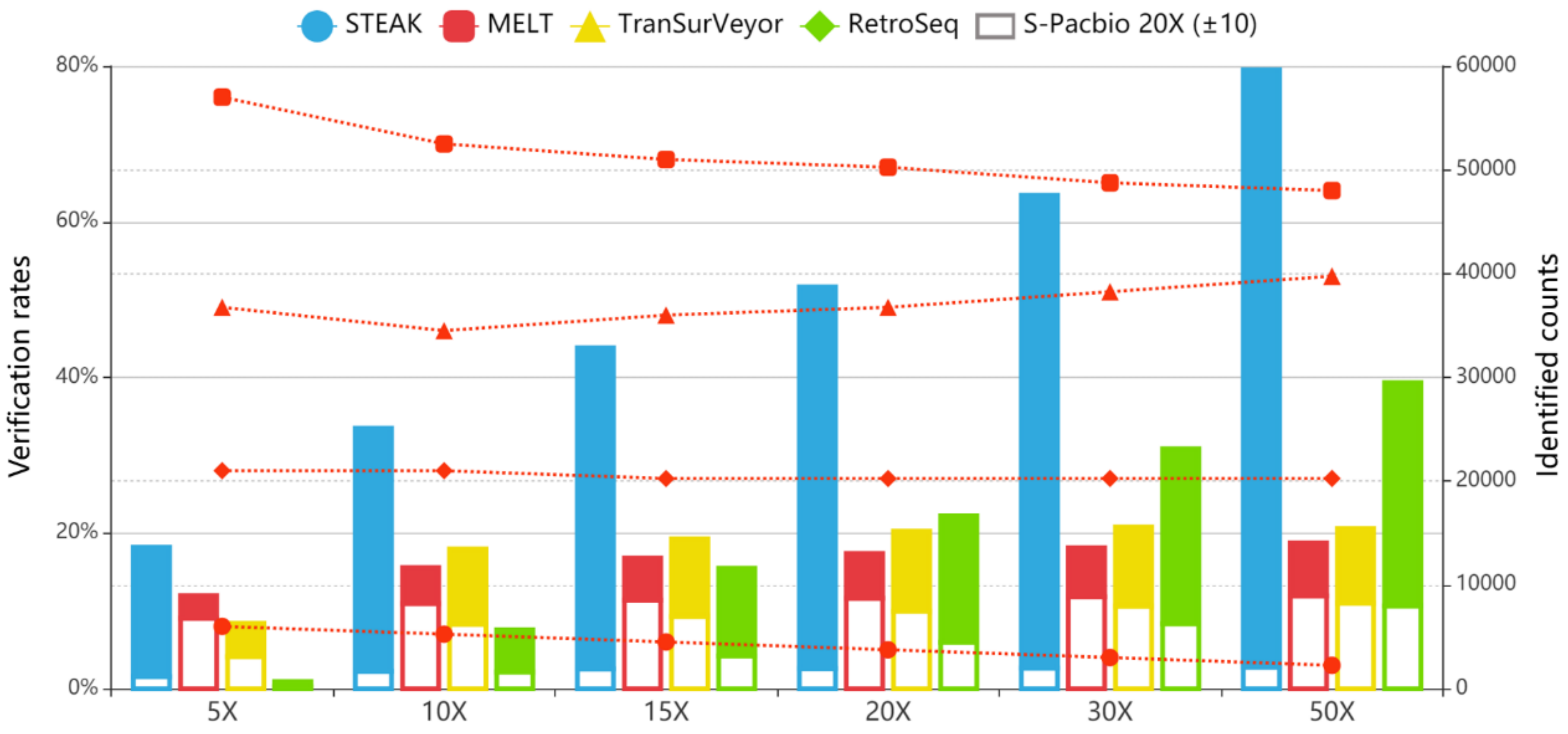

Supplementary Figure 18. The identified counts and verification rates of *Ref*+ ploySINEs are shown for different detection tools at varying sequencing depths. The blue, red, yellow, and green bars represent the counts identified by STEAK, MELT, TranSurVeyor, and RetroSeq, respectively. The blank bar represents the counts supported by polySINEs from PacBio. The lines represent the validation rates of each software by PacBio.

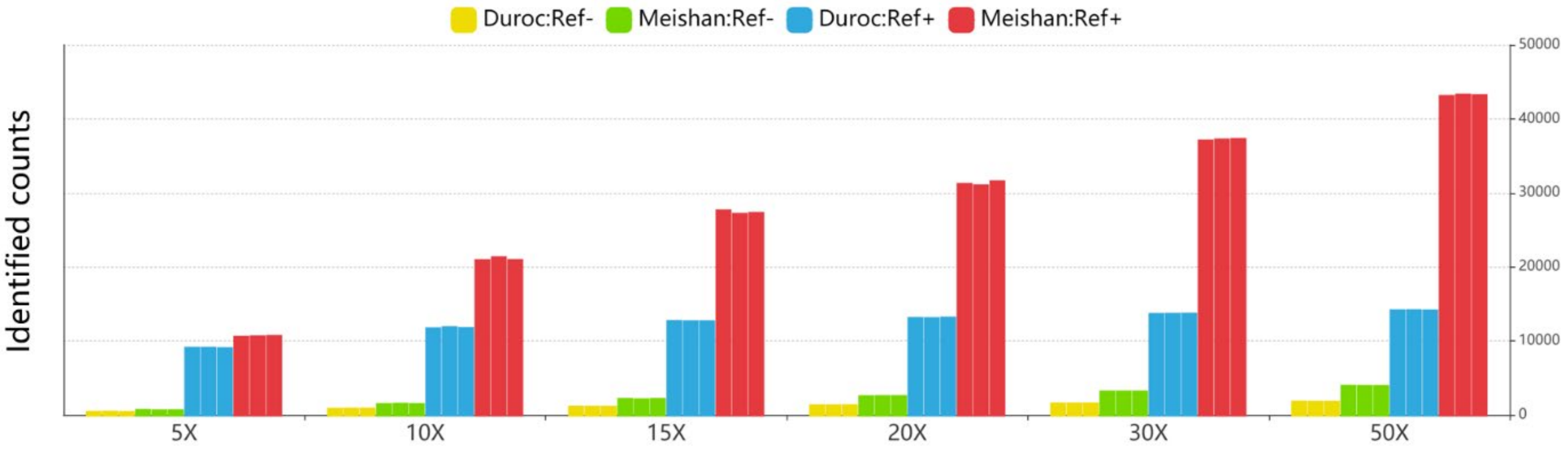

Supplementary Figure 19. The bar graph displays the counts of polySINEs identified in Meishan and Duroc pigs using MELT tools with varying sequencing depths. The x-axis represents sequencing depth, and the y-axis represents the sequencing identification number. The colors in the graph denote different variations among breeds.

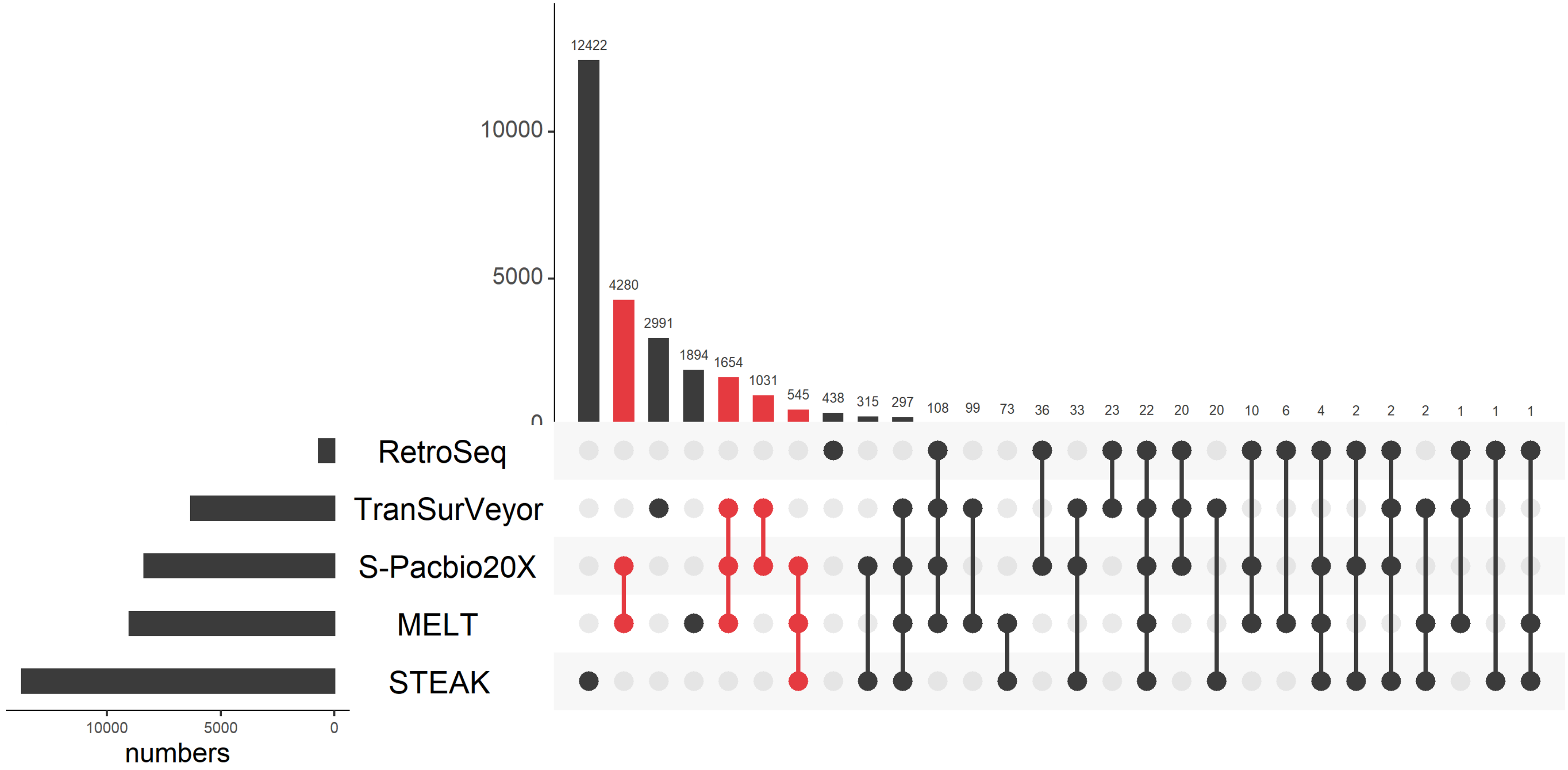

Supplementary Figure 20. Performance comparison of the detection tools for *Ref+* polySINEs at a sequencing depth of 5X. The y-axis in the upper bar plots represents the number of detected polySINEs. The red bar in the figure represents the software combination of the top 80% proportion of non-redundant polySINEs supported by PacBio data.

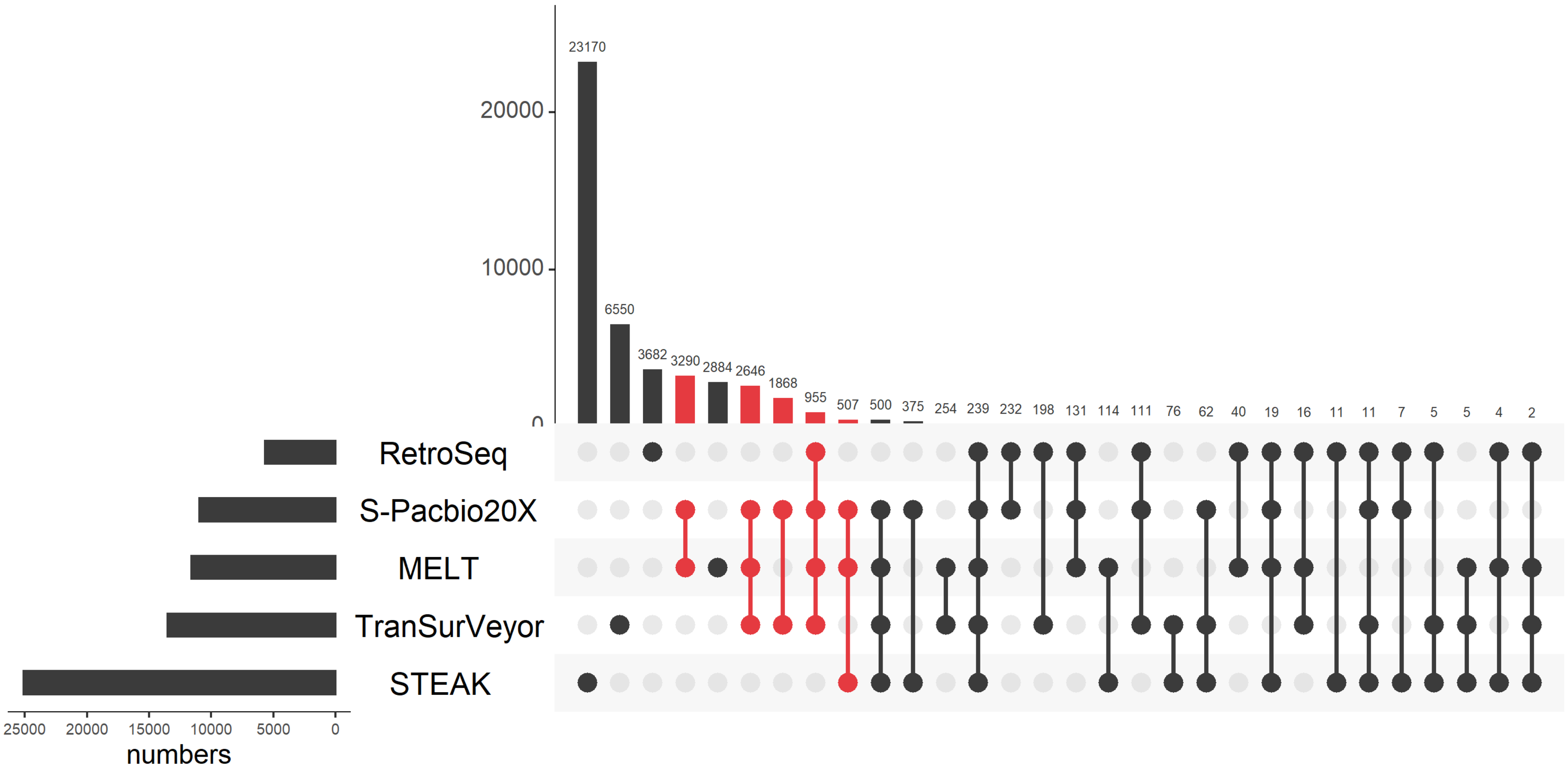

Supplementary Figure 21. Performance comparison of the detection tools for *Ref+* polySINEs at a sequencing depth of 10X. The y-axis in the upper bar plots represents the number of detected polySINEs. The red bar in the figure represents the software combination of the top 80% proportion of non-redundant polySINEs supported by PacBio data.

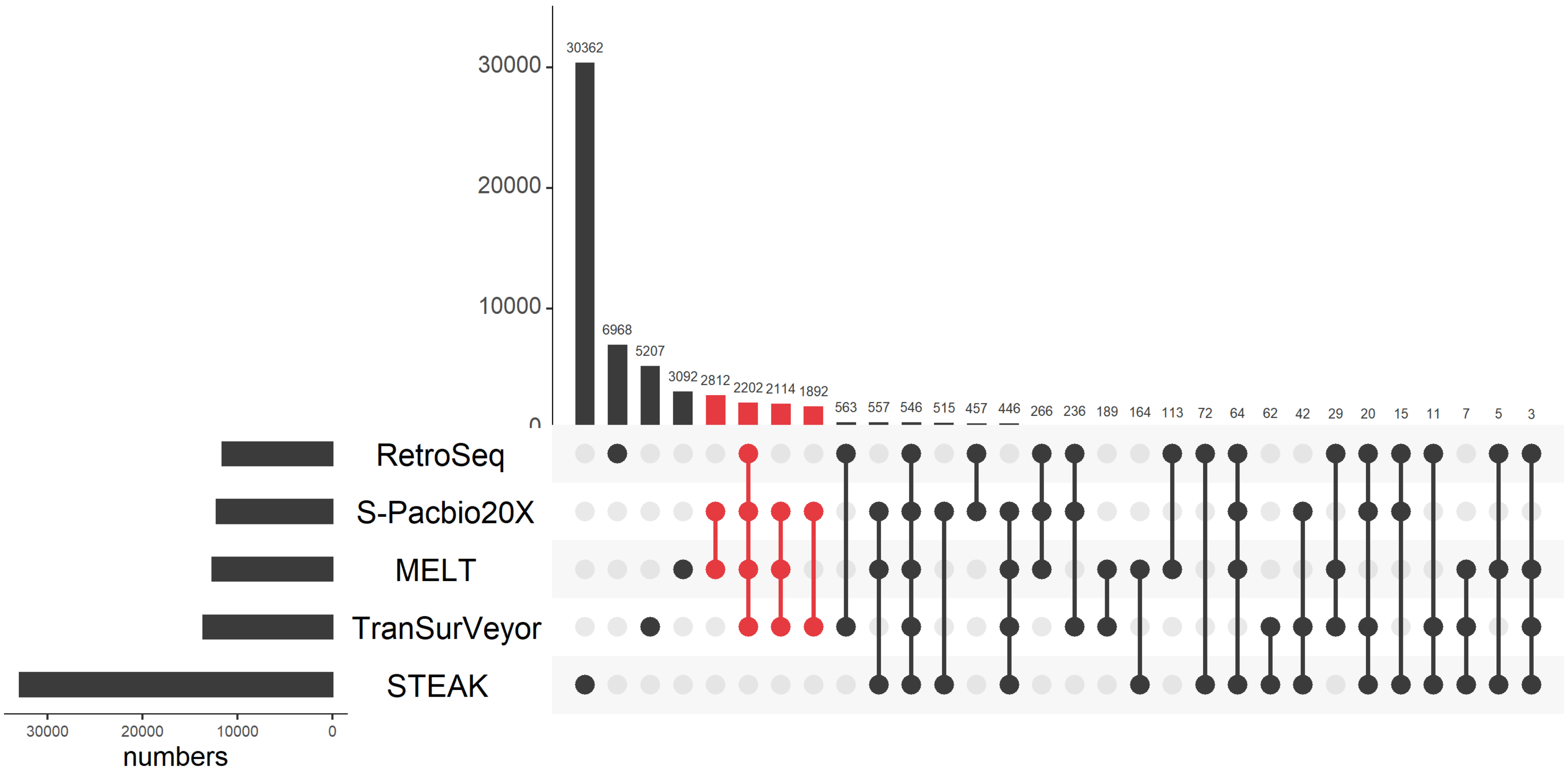

Supplementary Figure 22. Performance comparison of the detection tools for *Ref+* polySINEs at a sequencing depth of 15X. The y-axis in the upper bar plots represents the number of detected polySINEs. The red bar in the figure represents the software combination of the top 80% proportion of non-redundant polySINEs supported by PacBio data.

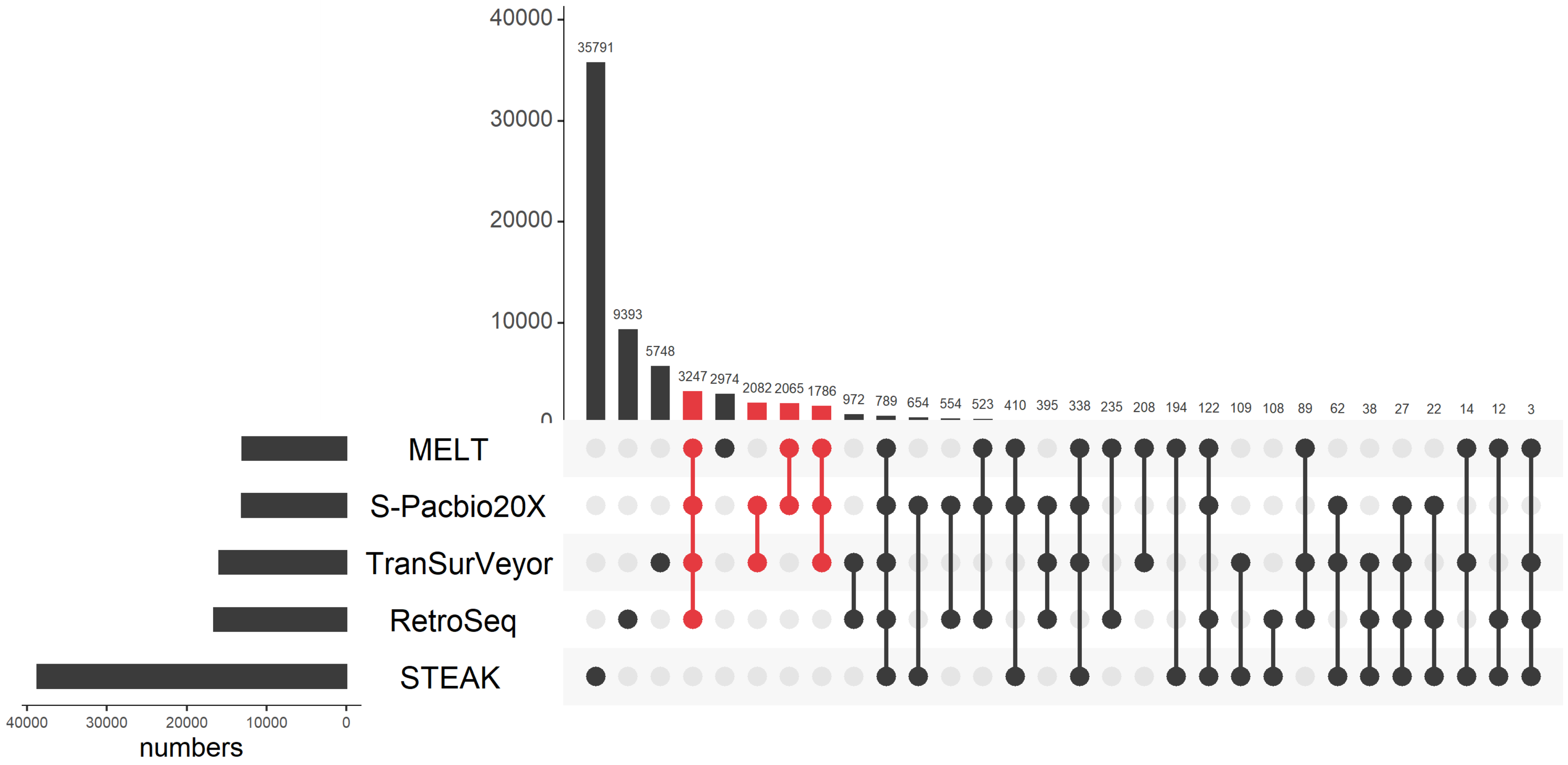

Supplementary Figure 23. Performance comparison of the detection tools for *Ref+* polySINEs at a sequencing depth of 20X. The y-axis in the upper bar plots represents the number of detected polySINEs. The red bar in the figure represents the software combination of the top 80% proportion of non-redundant polySINEs supported by PacBio data.

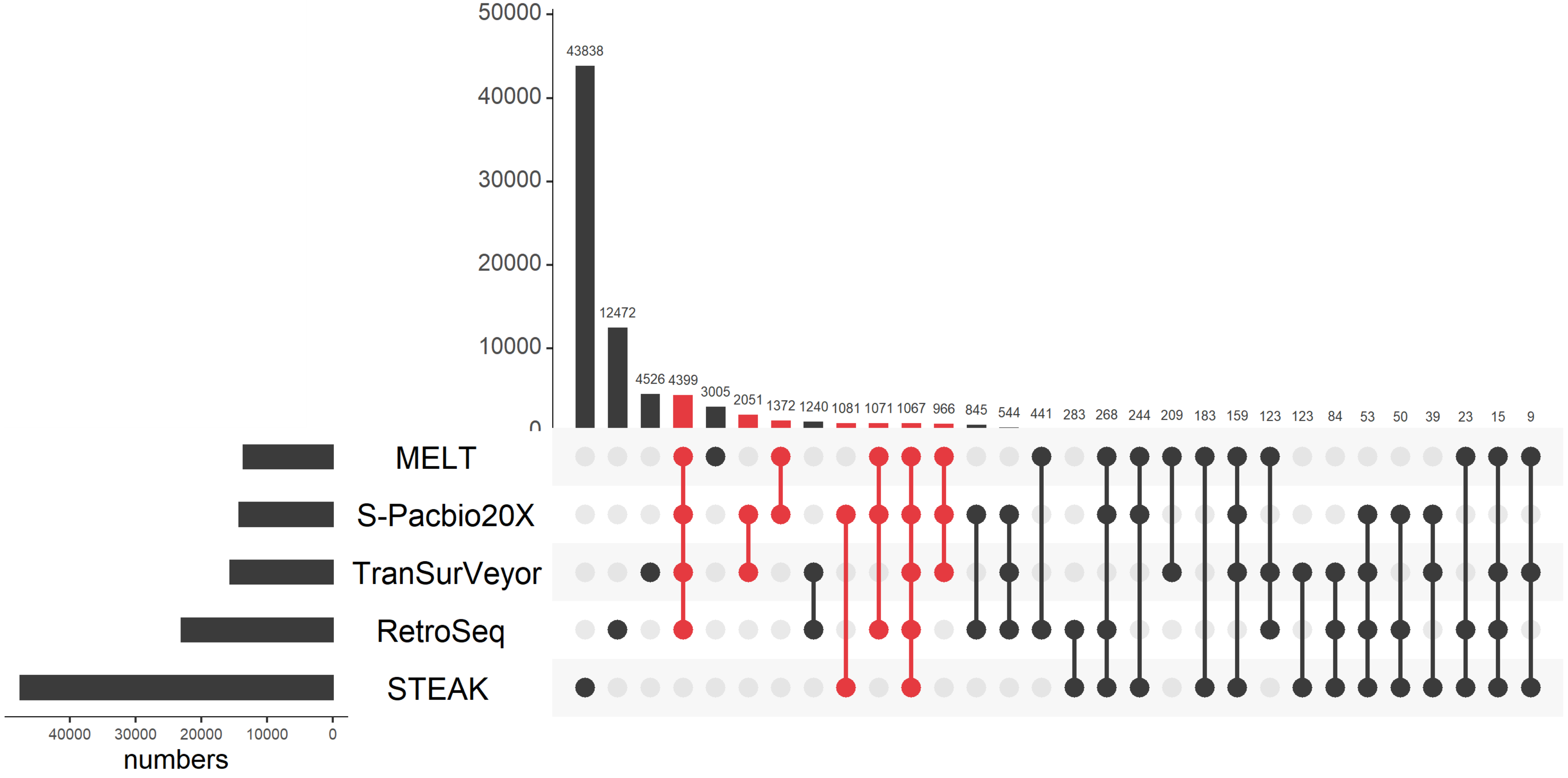

Supplementary Figure 24. Performance comparison of the detection tools for *Ref+* polySINEs at a sequencing depth of 30X. The y-axis in the upper bar plots represents the number of detected polySINEs. The red bar in the figure represents the software combination of the top 80% proportion of non-redundant polySINEs supported by PacBio data.

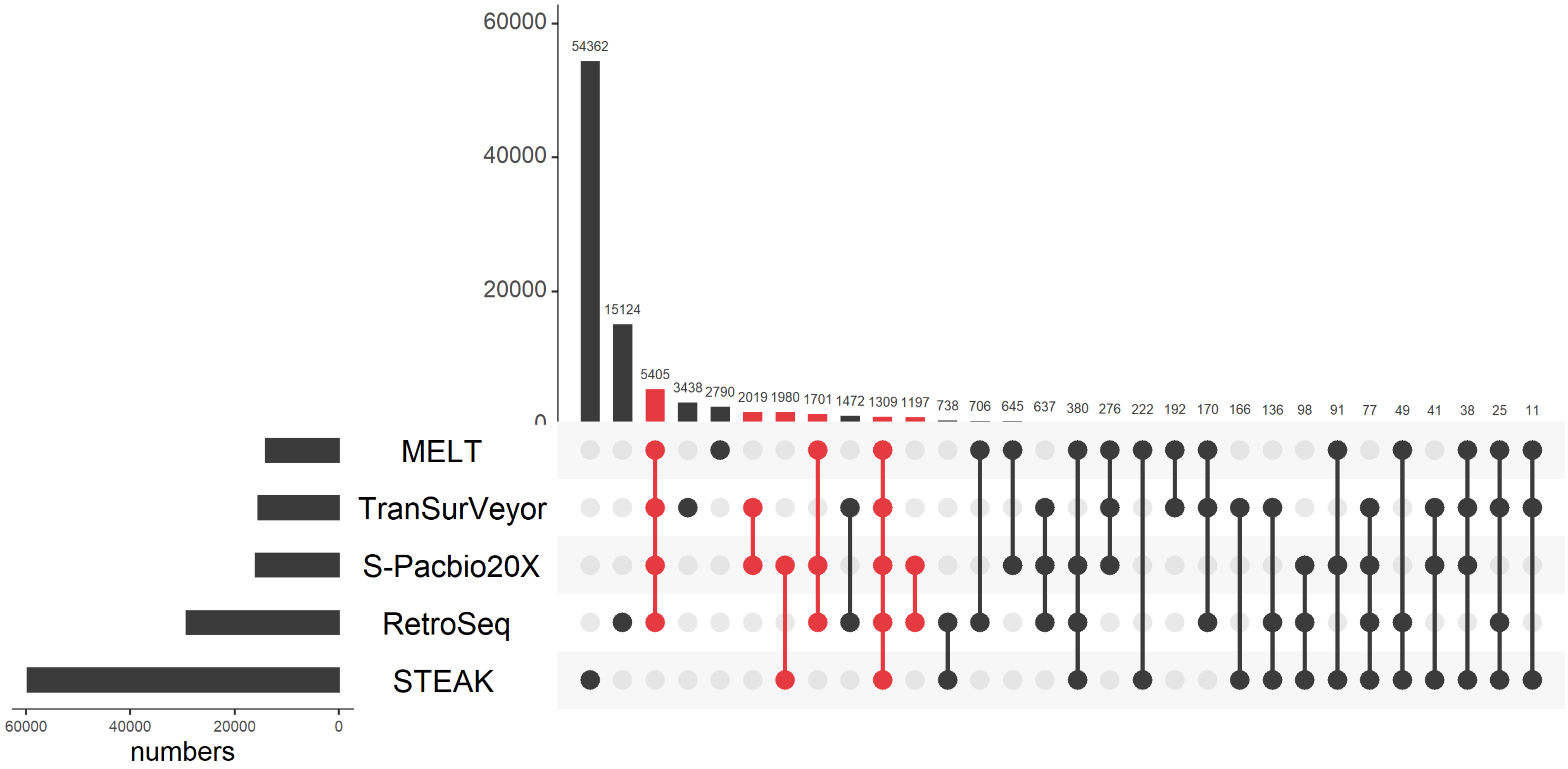

Supplementary Figure 25. Performance comparison of the detection tools for *Ref*+ polySINEs at a sequencing depth of 50X. The y-axis in the upper bar plots represents the number of detected polySINEs. The red bar in the figure represents the software combination of the top 80% proportion of non-redundant polySINEs supported by PacBio data.

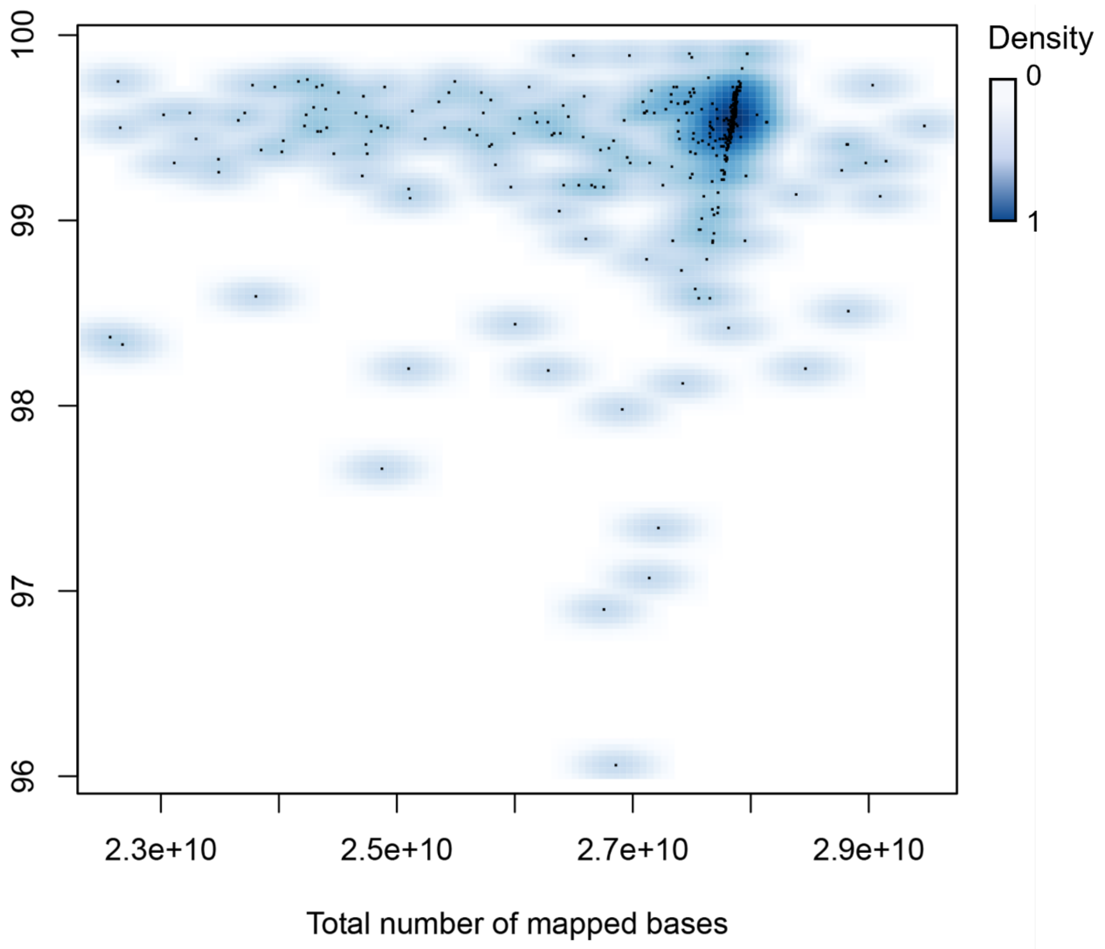

Supplementary Figure 26. Distribution of mapping rate and the total number of mapped bases for each individual. The x-axis represents the total number of mapped bases, while the y-axis represents the mapping rate.

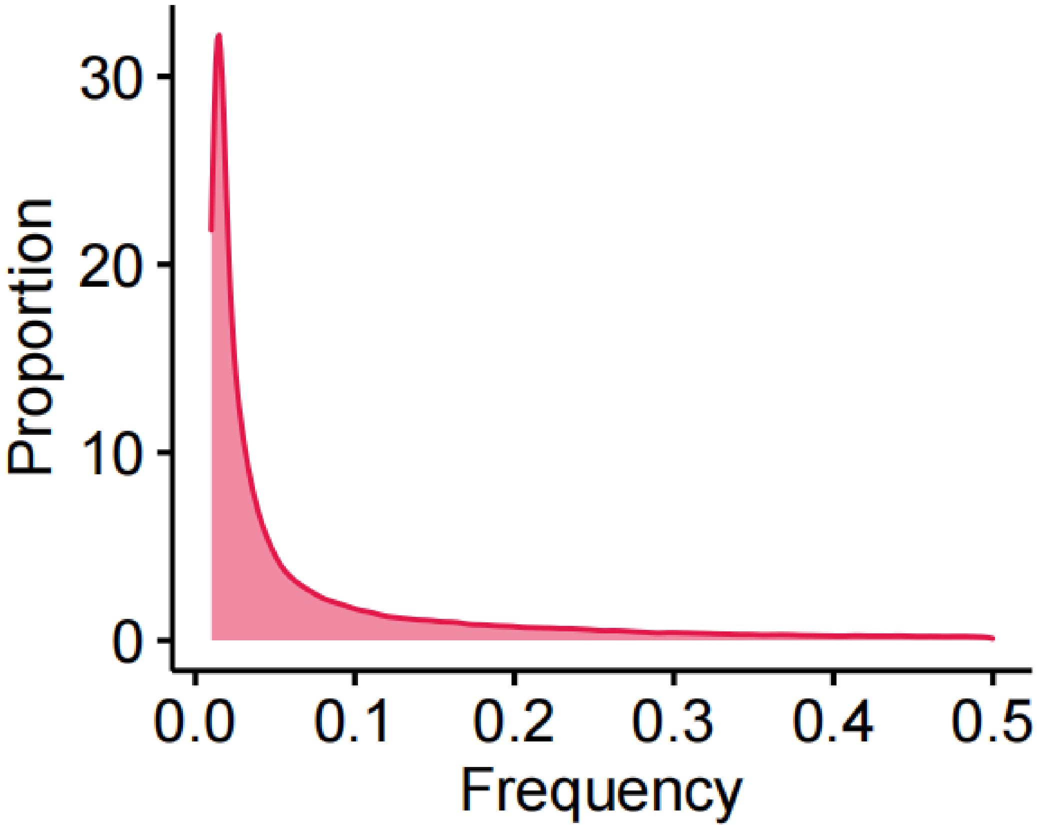

Supplementary Figure 27. Density distribution of polySINEs in the whole population. The x-axis represents the population frequency of polySINEs, while the y-axis represents the corresponding proportion.

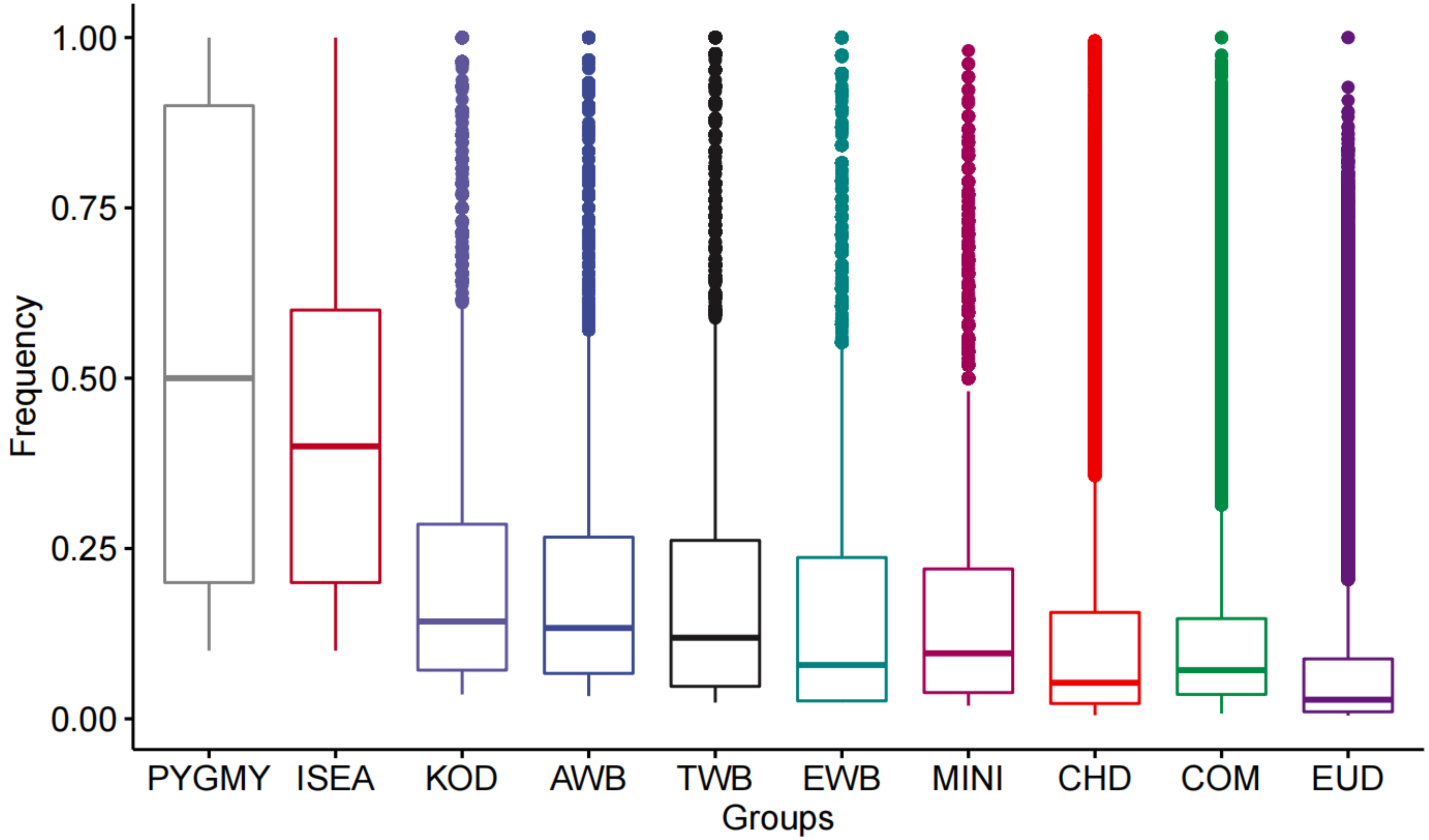

Supplementary Figure 28. The box plot shows the frequency distribution of polySINEs in different groups. The x-axis indicates the various groups, while the y-axis indicates the population frequency of polySINEs. The line inside the boxplots represents the median.

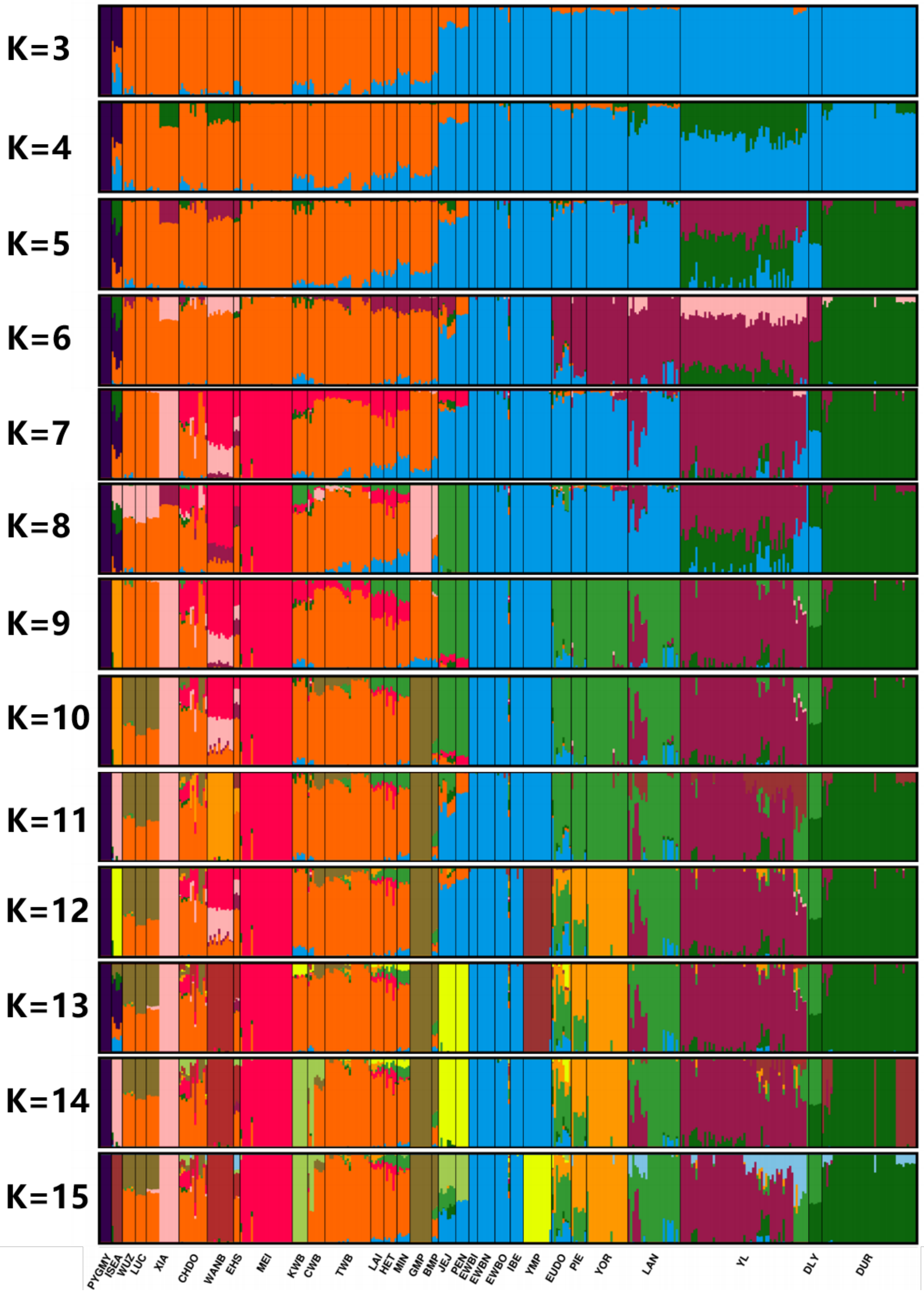

Supplementary Figure 29. Population structure based on polySINEs for 381 individuals when K varied from 3 to 15.

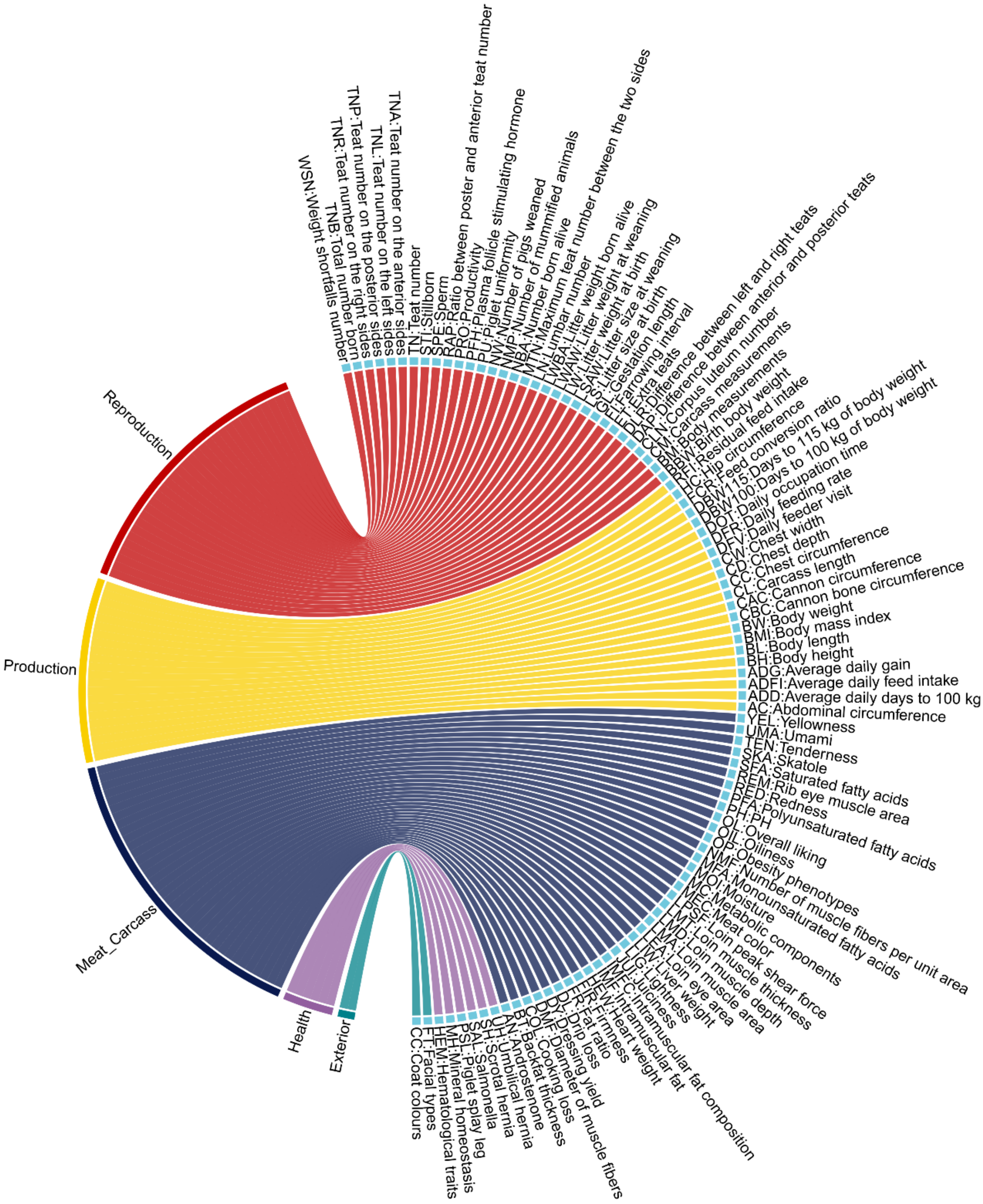

Supplementary Figure 30. Overviews of 79 published GWAS studies involve 97 complex traits. These include reproduction, production, meat and carcass, health, and exterior traits in pigs.

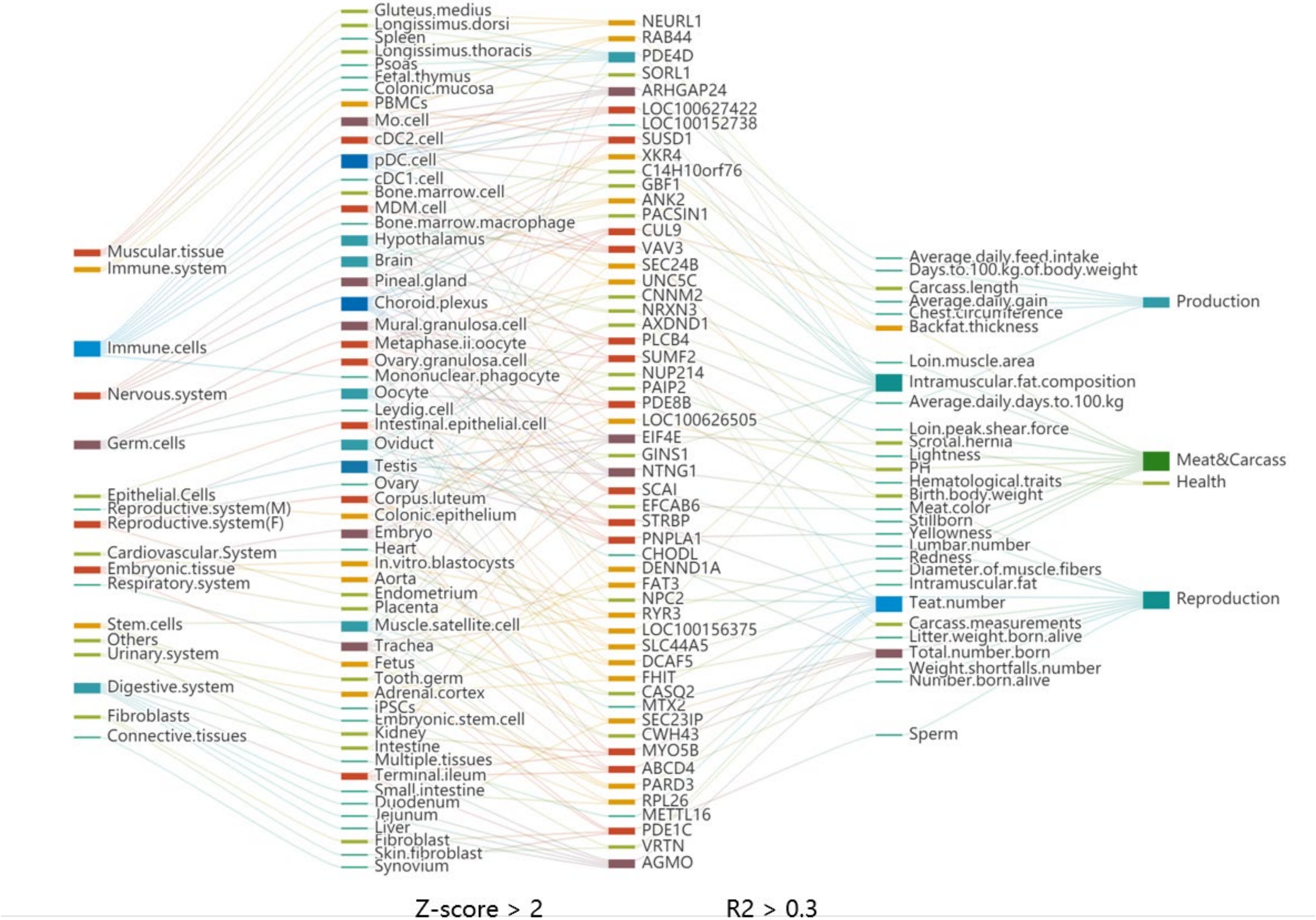

Supplementary Figure 31. Sankey plot represents the relationship among systems, tissues, genes, and traits.

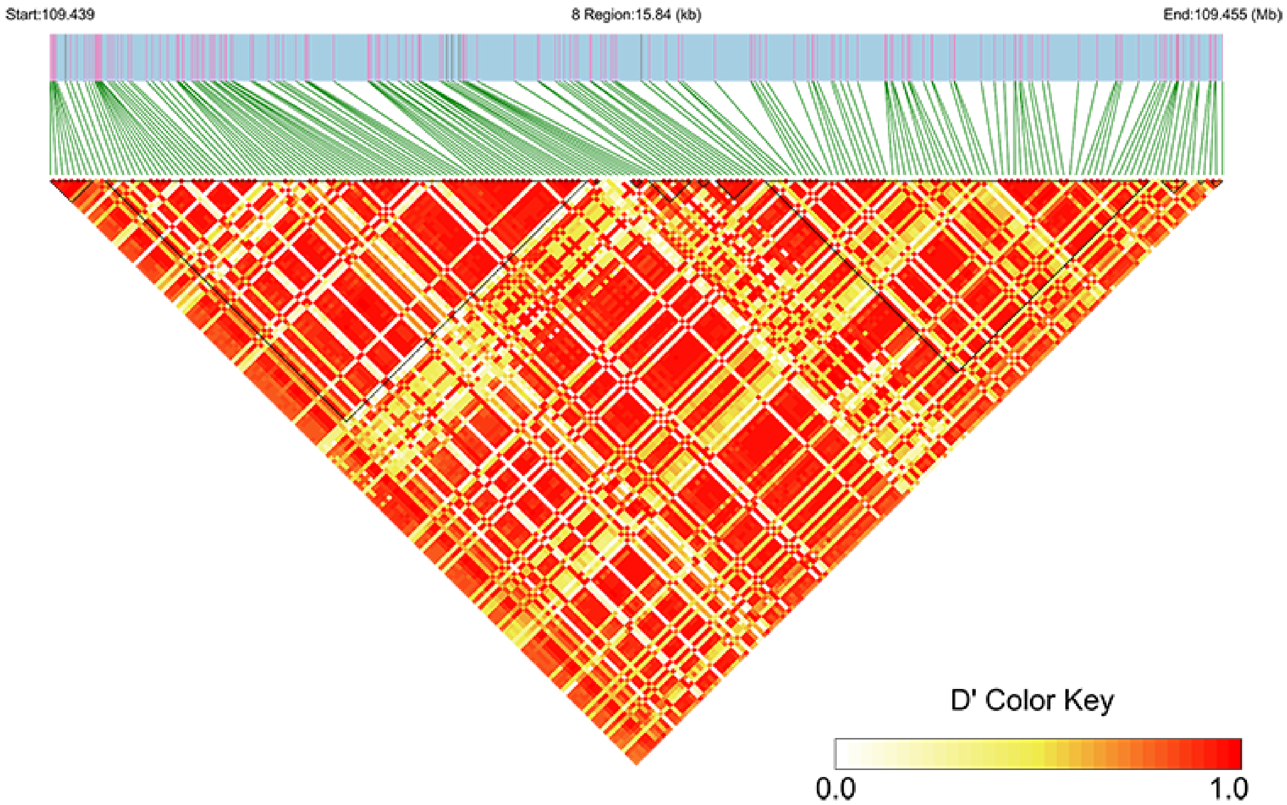

Supplementary Figure 32. The LD block of 15kb at chr8 from 109,439,023 to 109,454,866

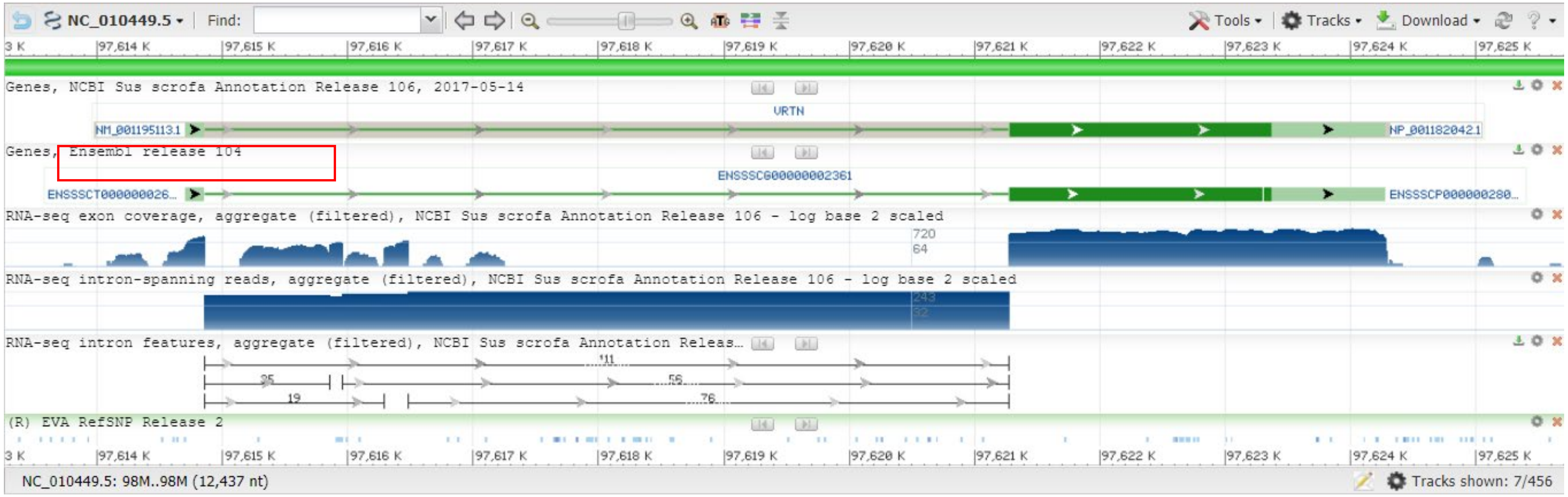

Supplementary Figure 33. NCBI annotation of *VRTN* gene and the RNA-seq exon coverage on the first exon of *VRTN*
