# Supplementary material for: Young SINEs in pig genomes impact gene regulation, genetic diversity, and complex traits
Source: Commun Biol. 2023 Aug 31;6:894. doi: 10.1038/s42003-023-05234-x (PMC10471783; doi:10.1038/s42003-023-05234-x)
Supplement: Supplementary file 3 — Description of Additional Supplementary Files [file 42003_2023_5234_MOESM3_ESM.pdf]

Description of Additional Supplementary Files

File name: Supplementary Data 1
Description: Details of 90 young SINE subfamilies.

File name: Supplementary Data 2
Description: The Z-scores and p-values for genomic and epigenomic feature enrichments.

File name: Supplementary Data 3
Description: 3,570 published RNA-seq samples from 52 tissues and 27 cell types.

File name: Supplementary Data 4
Description: 517 PCGs and 47 LncRNAs with SINE-associated transcripts perfectly matched.

File name: Supplementary Data 5
Description: Functional regions affected by SINE in four transcript categories.

File name: Supplementary Data 6
Description: Relationship between 13,872 PCGs and 40 modules.

File name: Supplementary Data 7
Description: Details of 374 individuals from 25 diverse populations.

File name: Supplementary Data 8
Description: The functional regions of 330 PCGs have the dimorphic SINEs with extreme Fst.

File name: Supplementary Data 9
Description: KEGG pathway for the 30 PCGs with the extreme Fst in the laboratory-inbred Bama Xiang pigs.

File name: Supplementary Data 10
Description: The 54 candidate genes potentially affected by T-dimorphic SINEs.

File name: Supplementary Data 11
Description: Three predicted SINE-associated transcripts for ANK2 and VRTN genes.

File name: Supplementary Data 12
Description: 16 whole genome bisulfite sequencing studies in pigs.

File name: Supplementary Data 13
Description: 20 small non-coding RNA studies in pigs.
