# Supplementary material for: Young SINEs in pig genomes impact gene regulation, genetic diversity, and complex traits
Source: Commun Biol. 2023 Aug 31;6:894. doi: 10.1038/s42003-023-05234-x (PMC10471783; doi:10.1038/s42003-023-05234-x)
Supplement: Supplementary file 5 — Reporting Summary [file 42003_2023_5234_MOESM5_ESM.pdf]

Corresponding author(s): Zhengguang Wang
Last updated by author(s): Aug 4, 2023

Reporting Summary

Nature Portfolio wishes to improve the reproducibility of the work that we publish. This form provides structure for consistency and transparency in reporting. For further information on Nature Portfolio policies, see our Editorial Policies and the Editorial Policy Checklist.

Statistics

For all statistical analyses, confirm that the following items are present in the figure legend, table legend, main text, or Methods section.

n/a	Confirmed	
☐	☒	The exact sample size (*n*) for each experimental group/condition, given as a discrete number and unit of measurement
☐	☒	A statement on whether measurements were taken from distinct samples or whether the same sample was measured repeatedly
☐	☒	The statistical test(s) used AND whether they are one- or two-sided<br>*Only common tests should be described solely by name; describe more complex techniques in the Methods section.*
☒	☐	A description of all covariates tested
☒	☐	A description of any assumptions or corrections, such as tests of normality and adjustment for multiple comparisons
☐	☒	A full description of the statistical parameters including central tendency (e.g. means) or other basic estimates (e.g. regression coefficient) AND variation (e.g. standard deviation) or associated estimates of uncertainty (e.g. confidence intervals)
☐	☒	For null hypothesis testing, the test statistic (e.g. *F*, *t*, *r*) with confidence intervals, effect sizes, degrees of freedom and *P* value noted<br>*Give P values as exact values whenever suitable.*
☒	☐	For Bayesian analysis, information on the choice of priors and Markov chain Monte Carlo settings
☐	☒	For hierarchical and complex designs, identification of the appropriate level for tests and full reporting of outcomes
☐	☒	Estimates of effect sizes (e.g. Cohen's *d*, Pearson's *r*), indicating how they were calculated

Our web collection on statistics for biologists contains articles on many of the points above.

Software and code

Policy information about availability of computer code

Data collection	Five pig MNase-seq samples were downloaded from NCBI (BioProject ID PRJNA417273) representing brain, heart, kidney, liver, and muscle tissues. H3K4me1, H3K4me3, H3K27ac, and H3K27me3 in 14 tissues were downloaded from NCBI under BioProject ID PRJNA762083. In addition, we downloaded one H3K9me3 sample (BioProject ID PRJNA152995), and four H3K36me3 (BioProject ID PRJNA529704) and H3K27me3 (BioProject ID PRJEB31243) samples each. Chromatin states of pigs were analyzed in 14 different tissues, which were downloaded from (http://genome.ucsc.edu/s/zhypan/susScr11_15_state_14_tissues_new).<br>The MeDIP-Seq used in this study included 80 samples that were downloaded from NCBI under BioProject ID PRJNA143661. Whole Genome Bisulfite Sequencing (WGBS) data used in this study included 246 samples from 10 tissues, downloaded from 16 studies (Table S11). We collected data from 177 samples across 20 pig small non-coding RNA studies (Table S12). We downloaded two PacBio long-read isoform sequencing data sets from NCBI (BioProject ID PRJCA000349, pooled 38 samples, and PRJNA351265). Transcriptome data from 52 tissues and 27 types of cells were downloaded from NCBI in Table S2.
Data analysis	All software used in this study is open source.

For manuscripts utilizing custom algorithms or software that are central to the research but not yet described in published literature, software must be made available to editors and reviewers. We strongly encourage code deposition in a community repository (e.g. GitHub). See the Nature Portfolio guidelines for submitting code & software for further information.

Data

Policy information about availability of data

All manuscripts must include a data availability statement. This statement should provide the following information, where applicable:

- Accession codes, unique identifiers, or web links for publicly available datasets
- A description of any restrictions on data availability
- For clinical datasets or third party data, please ensure that the statement adheres to our policy

To make our research data accessible to readers, we provide detailed information on data availability. This includes the types and format of data, data collection procedures, data quality control, and data sources. By doing this, we aim to enable future research and allow others in the scientific community to build upon and replicate our findings.

Research involving human participants, their data, or biological material

Policy information about studies with human participants or human data. See also policy information about sex, gender (identity/presentation), and sexual orientation and race, ethnicity and racism.

Reporting on sex and gender	Uninvolved
Reporting on race, ethnicity, or other socially relevant groupings	Uninvolved
Population characteristics	Uninvolved
Recruitment	Uninvolved
Ethics oversight	Uninvolved

Note that full information on the approval of the study protocol must also be provided in the manuscript.

Field-specific reporting

Please select the one below that is the best fit for your research. If you are not sure, read the appropriate sections before making your selection.

☒ Life sciences ☐ Behavioural & social sciences ☐ Ecological, evolutionary & environmental sciences

For a reference copy of the document with all sections, see nature.com/documents/nr-reporting-summary-flat.pdf

Life sciences study design

All studies must disclose on these points even when the disclosure is negative.

Sample size	Sample size depends on the number of samples collected
Data exclusions	Downloaded samples that cannot be analyzed because they are incomplete will be excluded
Replication	Uninvolved
Randomization	Uninvolved
Blinding	Uninvolved

Reporting for specific materials, systems and methods

We require information from authors about some types of materials, experimental systems and methods used in many studies. Here, indicate whether each material, system or method listed is relevant to your study. If you are not sure if a list item applies to your research, read the appropriate section before selecting a response.

Materials & experimental systems

n/a	Involved in the study
☒	☐ Antibodies
☒	☐ Eukaryotic cell lines
☒	☐ Palaeontology and archaeology
☒	☐ Animals and other organisms
☒	☐ Clinical data
☒	☐ Dual use research of concern
☒	☐ Plants

Methods

n/a	Involved in the study
☒	☐ ChIP-seq
☒	☐ Flow cytometry
☒	☐ MRI-based neuroimaging
